# Comparative profiles of lubiprostone, linaclotide, and elobixibat for chronic constipation: a systematic literature review with meta-analysis and number needed to treat/harm

**DOI:** 10.1186/s12876-023-03104-8

**Published:** 2024-01-02

**Authors:** Satish S. Rao, Noriaki Manabe, Yusuke Karasawa, Yuko Hasebe, Kazutaka Nozawa, Atsushi Nakajima, Shin Fukudo

**Affiliations:** 1 Division of Gastroenterology and Hepatology, Medical College of Georgia, Augusta University, Augusta, GA USA; 2 Division of Endoscopy and Ultrasonography, Department of Clinical Pathology and Laboratory Medicine, Kawasaki Medical School General Medical Center, Okayama, Japan; 3 Medical Affairs, Viatris Pharmaceuticals Japan Inc, Tokyo, Japan; 4 Department of Gastroenterology and Hepatology, Yokohama City University Graduate School of Medicine, Yokohama, Japan; 5 Department of Behavioral Medicine, Tohoku University Graduate School of Medicine, Sendai, Japan

**Keywords:** Constipation, Lubiprostone, Linaclotide, Elobixibat, Meta-analysis

## Abstract

**Objective:**

To comprehensively evaluate the efficacy, safety, patient symptoms, and quality-of-life (QoL) of lubiprostone, linaclotide, and elobixibat as treatment for chronic constipation (CC).

**Design:**

Systematic literature review (SLR) and meta-analysis (MA). Literature searches were conducted on PubMed and Embase using the Ovid platform.

**Methods:**

SLR including randomized controlled trials (RCTs) and observational studies was conducted to identify the overall efficacy and safety of lubiprostone, linaclotide, and elobixibat. Thereafter, MA was performed using only RCTs. The number needed to treat (NNT) and number needed to harm (NNH) analyses were additionally conducted.

**Primary and secondary outcome measures:**

The primary outcome was efficacy regarding change in spontaneous bowel movements. Secondary outcomes included safety, constipation-related symptoms, and QoL.

**Results:**

Twenty-four studies met the inclusion criteria for the SLR: 17 RCTs, 4 observational studies, and 3 single-arm trials. Feasibility assessment for the MA resulted in 14 studies available for safety data analysis, and 8 available for efficacy analysis, respectively. Three drugs showed similar efficacy in the MA and NNT analysis. However, the NNH analysis revealed distinct safety profiles: lubiprostone, linaclotide, and elobixibat were linked to the highest risk of nausea, diarrhea, and abdominal pain, respectively.

**Conclusion:**

The current study provides an updated overview of the efficacy, safety, patient symptoms, and QoL of the three drugs with different mechanisms of action for CC treatment.The findings could help physicians adopt an individualized approach for treating patients with CC in clinical practice.

**Supplementary Information:**

The online version contains supplementary material available at 10.1186/s12876-023-03104-8.

## Introduction

Constipation is a common gastrointestinal disorder characterized by persistently difficult, incomplete, or infrequent bowel movements [1]. Chronic constipation (CC), which is generally defined as symptoms persisting for at least 3 months, comprises primary (functional or idiopathic) or secondary constipation associated with metabolic abnormalities, neurological disorders, psychological disorders, lifestyle factors, or the intake of medications such as opiates and antidepressants [2]. The Rome IV criteria for diagnosing functional constipation (FC) require the presence of at least 2 of the following symptoms in at least 25% of defecations: (a) fewer than 3 spontaneous bowel movements (SBMs) per week; (b) straining; (c) lumpy or hard stools; (d) sensation of incomplete evacuation; (e) sensation of anorectal obstruction; or (f) manual maneuvers to facilitate defecations.

The global prevalence of Rome IV FC was 11.7% [3] and that of chronic idiopathic constipation (CIC) was found to be 14% [4]. It is estimated that approximately 35 million adult Americans have CIC and that 16 of 100 adults have symptoms of constipation [5]. A systematic review by Peppas et al. revealed the prevalence of constipation to be 16.6% among the general European population [4]. The prevalence of FC according to the Rome IV criteria in Japan was 16.6% (95% confidence interval 15.1–18.0%) [3]. During a routine medical check-up, Rome IV FC was detected in 2.1% of patients, with female sex, lack of exercise, insufficient sleep, and eating faster being risk factors [6]. Furthermore, the elderly population reports a higher incidence of constipation compared with the younger population [7]. Existing evidence suggests a lower health-related quality-of-life (HRQoL) among patients with constipation compared with healthy individuals. Management of constipation improves HRQoL. Additionally, constipation exerts a significant cost burden on patients, with both diagnosis and treatment being major cost drivers [8].

Regarding pharmacological treatment, a few traditional laxatives have been widely used. Stimulant laxatives are broadly classified into anthraquinone laxatives (sennoside, etc.) and diphenyl laxatives (bisacodyl, picosulfate, etc.), which are recommended for short-term use by the American Gastroenterological Association guidelines [9]. Their long-term use may be associated with a risk of tolerance, which can lead to refractory constipation. Bulking laxatives (bran, psyllium, etc.) and osmotic laxatives (polyethylene glycol, lactulose, etc.) are also commonly used [10]. However, it has been indicated that patients as well as health care professionals reported dissatisfaction with relief from constipation symptoms after using fiber supplements, OTC laxatives, or prescription drugs, despite a wide array of treatment options being available [11, 12].

To overcome this challenge, several agents have been developed that increase intestinal secretion of fluid and electrolytes by different mechanisms. Prosecretory agents (secretagogues) such as lubiprostone and linaclotide stimulate intestinal fluid secretion by somewhat different mechanisms, and the ileal bile acid transporter inhibitor, elobixibat, increases intestinal secretion and transit. Lubiprostone, a bicyclic fatty acid derived from prostaglandin E1, stimulates the chloride channel type 2 (ClC-2) located on the intestinal epithelium’s apical surface [13]. As a consequence, the intestinal lumen experiences an influx of chloride and water, which accelerates the passage through both the small and large intestines. The activation of guanylate cyclase type C reception by linaclotide, the second secretagogue for CIC to be approved by the Food and Drug Administration (FDA), results in an elevation of cyclic guanosine monophosphate [14]. This increases water secretion into the intestinal lumen, which in turn accelerates intestinal transit, by stimulating chloride secretion. By impeding bile acid absorption, elobixibat increases bile acid concentration in the gastrointestinal tract, which facilitates intestinal transit and softens feces [15]. However, there is limited knowledge regarding the appropriate selection of these novel medical therapies. Although several systematic reviews have been conducted on the efficacy of these agents [16–18], there are limited data summarizing outcomes other than the frequency of defecation. We believe that a comprehensive evaluation covering the efficacy, safety, and effects of these agents on patient symptoms and QoL would help physicians formulate suitable treatment strategies. Therefore, we conducted a comprehensive systematic literature review (SLR) and meta-analysis (MA) to understand the role of new therapies (lubiprostone, linaclotide, and elobixibat) in CC treatment, focusing not only on their efficacy but also their safety, effect on symptoms, and QoL. In addition, we calculated the number needed to treat (NNT) and the number needed to harm (NNH) for each medication.

## Methods

### Study design and search strategy

**Fig. 1:**
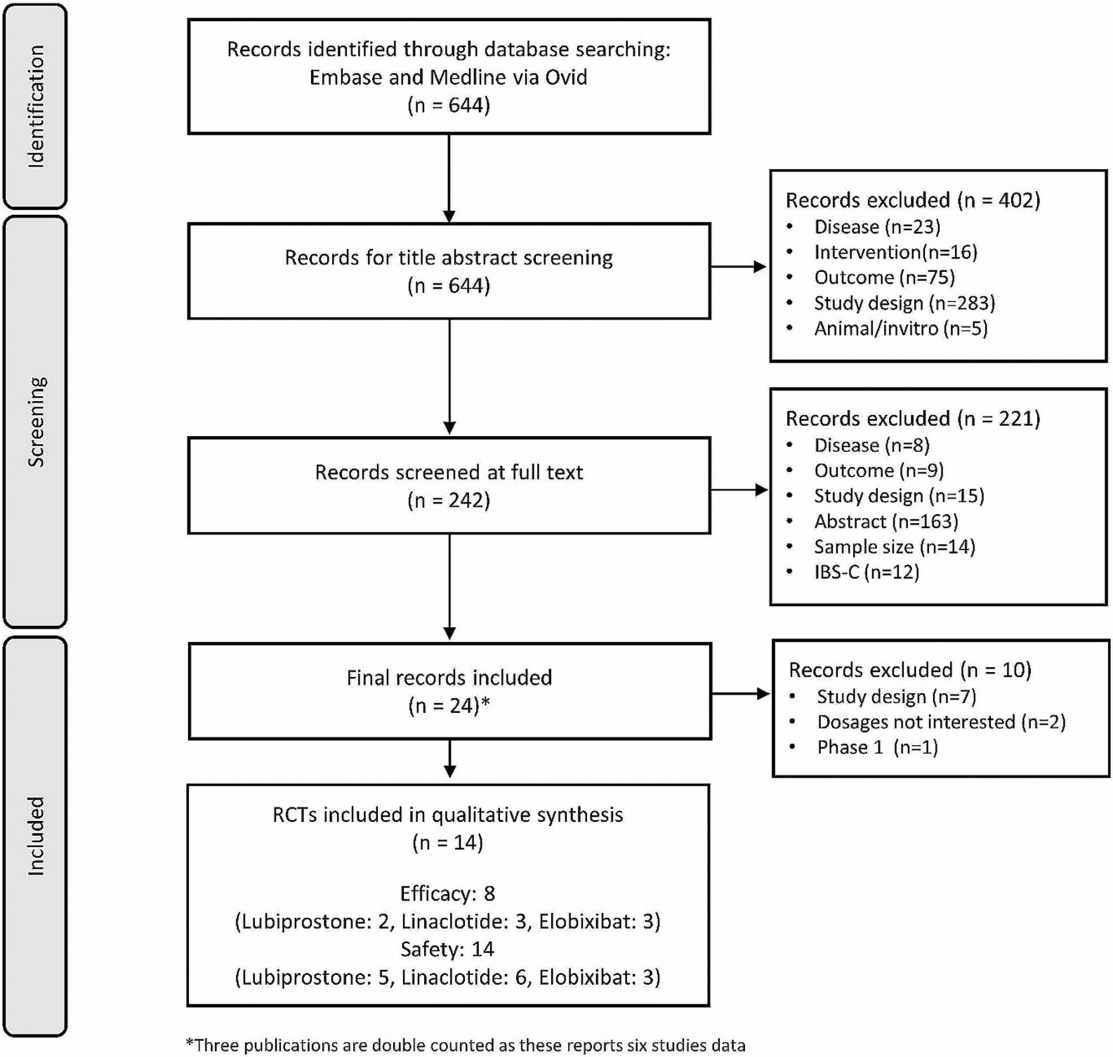
Literature search and study selection

We performed an SLR and MA to compare the approved dose of lubiprostone 48 mcg, linaclotide 145 mcg or 500 mcg, and elobixibat 10 mg or 15 mg following Preferred Reporting Items for Systematic Reviews and Meta-Analyses (PRISMA) guidelines [19]. The SLR was conducted on PubMed and Embase using the Ovid platform on September 8, 2020. In addition, a few recent and relevant SLRs and pooled studies were also cross-checked to identify any other appropriate studies. The search strategy used is presented in Fig. 1. During the feasibility assessment for the MA, another 10 studies were excluded, and, finally, 14 studies were included for the MA. All 14 studies were included in the safety analysis. A clear definition of the study participants, interventions, comparison groups, outcomes, and study types of interest was attempted as per the Population, Intervention, Comparison, Outcomes and Study (PICOS) criteria (supplementary Table S1). There was no limitation regarding geographic scope or language.

All studies retrieved from the literature search were evaluated for relevance against the eligibility criteria, and a final selection of studies to inform the reviews was made. At the end of the selection process, a list of all the included studies was generated.

### Data extraction

Data extraction was performed for all the included studies by 1 reviewer, and a quality check was performed by another reviewer. The objective of the data extraction was to capture sufficient detailed information on the patient population (inclusion criteria, sample size, patient demographics, and baseline disease characteristics), interventions (treatment class, dose, and treatment duration), outcomes (definitions, measurement methodology, and follow-up duration), and study design (use of randomization, blinding, and number of treatment arms).

The studies were assessed for quality based on parameters such as the generalization of the study methodology, characteristics of patients, treatment group(s), outcome details, and reporting of results. The sample size in each study for different populations was greater than 100. Most of the included studies were double-blind, placebo-controlled randomized controlled trials (RCTs). Most RCTs reported data for 1 week, followed by other timepoints, such as 2, 3, 4, 8, and 12 weeks. A few studies also reported data for 24, 26, 36, and 52 weeks.

### Quality assessment

#### Randomized controlled trials

The Cochrane risk of bias version 2 tool (RoB 2) was used to assess the risk of bias in RCTs [20]. It is structured into a fixed set of domains of bias, focusing on various aspects of trial design, conduct, and reporting. A series of questions is present within each domain to elicit information about the features of the trial related to the risk of bias. Based on the answer to each question, a proposed judgment about the risk of bias arising from each domain is generated by an algorithm. The judgment can be a “low” or “high” risk of bias or can be expressed as “some concerns.” In this review, a trial was considered high quality if it had a low risk of bias across all domains.

#### Observational and single-arm trials

The Newcastle–Ottawa Scale (NOS) was developed jointly by the University of Newcastle (Australia) and the University of Ottawa (Canada) to evaluate the quality of non-randomized studies to be included in systematic reviews [21]. Included studies were assessed based on 3 domains: selection, comparability, and outcome/exposure. The maximum possible score for each study was 9; if any individual component is not applicable, the maximum score decreases. Each study was graded as high, medium, or low quality. If the maximum NOS score was 9, the study scores were 8–9, 6–7, and < 6, marked as high, medium, and low quality, respectively. If the maximum NOS score was 8, the study scores were 7–8, 5–6, and < 5, marked as high, medium, and low quality, respectively.

### Outcome assessment

#### Efficacy analysis

The primary outcomes studied were SBMs. This included SBM frequency, change from baseline in SBM frequency, SBM in ≤ 24 h, the response rate, and the time to first SBM. The secondary outcomes were stool consistency, straining severity, abdominal bloating, abdominal pain/discomfort, and constipation severity. We defined SBM frequency at Week 1 as an efficacy outcome for MA. SBM frequency was used as a primary efficacy endpoint in most trials regarding constipation; hence, the study focused on the same endpoint. Further, all included studies in the MA except for one reported SBM frequency at Week 1 as the primary efficacy endpoint.

#### Safety analysis

The safety data included an analysis of adverse events (AEs), such as diarrhea, nausea, abdominal pain, and vomiting. Diarrhea was defined as a safety outcome for the MA.

#### Quality-of-life (QoL) analysi*s*

QoL data were obtained from the 36-Item Short Form Survey (SF-36) subscale (physical function, bodily pain, general health, vitality, role-emotional) [22], patient assessment of constipation-quality-of-life (PAC-QoL) score and subscales (physical discomfort, psychosocial discomfort, worries and concern, and satisfaction) [23], the Japanese version of the PAC-QoL (JPAC-QoL) score and subscales (physical discomfort, psychosocial discomfort, worries and concerns and satisfaction) [24], or the Japanese version of irritable bowel syndrome quality of life questionnaire (IBS-QoL) and subscales (dysphoria, interference with activity, body image, health worry, food avoidance, social reaction, sexual, relationships) [25].

### Meta-analysis feasibility assessment

A feasibility assessment was conducted to identify studies eligible for the MA. We compared the distribution of treatments, outcomes, study designs, and patient characteristics, which could act as potential effect modifiers of treatment or confounding variables. We also evaluated data availability per outcome of interest for the analysis.

### Sensitivity analysis

Across the included studies, very limited data for disease duration was available. Additionally, none of the included studies reported comorbidity data. However, other patient characteristics, such as age, sex, height, body mass index, and race/ethnicity, were similar. Since the sample sizes varied across studies, a sensitivity analysis was carried out to verify the effect of the sample size on the pooled estimate for diarrhea. For lubiprostone, across the included studies, only 1 study had a lower sample size (n = 32–33) compared with other studies (n = 42–122). Similarly, for linaclotide, 1 study had a higher sample size (n = 401–411) compared with other studies (n = 153–217). We excluded these studies from the analysis and assessed the effect of high and low sample sizes on the overall effect estimate.

### Statistical analysis

All calculations were performed according to the Cochrane handbook [26]. The formulas used in the calculation are presented below:

Standard error (SE) calculation using a 95% confidence interval (CI):

$$ \text{S}\text{E} = (95\text{\%} \text{C}\text{I} \text{u}\text{p}\text{p}\text{e}\text{r}- 95\text{\%} \text{C}\text{I}\text{l}\text{o}\text{w}\text{e}\text{r})/ (2 \times1.96)$$

SE calculation using standard deviation (SD):

$$ \text{S}\text{E}\hspace{0.17em}=\hspace{0.17em}\text{S}\text{D}/\surd \text{n}$$

Data presented in graphs for the 95% CI, SE, and responder rate were extracted using WebPlotDizitizer.

### Number needed to treat (NNT), and number needed to harm (NNH)

The NNT and NNH are useful measures for clinicians to understand a treatment’s potential for benefit and harm, respectively. NNT indicates how many patients need to be treated with an intervention for 1 patient to experience a favorable outcome. NNH details how many patients need to be treated with a particular intervention for 1 patient to have an AE. The NNH is a reciprocal of the change in absolute risk. NNT and NNH were calculated using the following formulas [27–29]:

$$ \text{N}\text{N}\text{T}\hspace{0.17em}=\hspace{0.17em}1/\text{A}\text{R}\text{R}\hspace{0.17em}=\hspace{0.17em}1/{\text{S}}_{\text{t}} - {\text{S}}_{\text{c}}$$

$$ \text{N}\text{N}\text{H}\hspace{0.17em}=\hspace{0.17em}1/\text{A}\text{R}\text{I}\hspace{0.17em}=\hspace{0.17em}1/{\text{S}}_{\text{t}} - {\text{S}}_{\text{c}},$$

where ARR: Absolute risk reduction; ARI: Absolute risk increase; S_t_: Event rate in treatment group; S_c_: Event rate in control group.

### Data analysis

The mean difference (MD) and risk ratio (RR) were used as effect estimates for continuous and dichotomous outcomes, respectively. Both I^2^ statistics and the χ^2^ test were used to evaluate heterogeneity. I^2^ statistics values vary between 0 and 100; I^2^ = 0 indicates the presence of heterogeneity due to a sampling error, and I^2^ = 100% indicates true heterogeneity between the studies [30]. The I^2^ statistic with a cut-off of ≥ 50% and the χ^2^ test with *p* < 0.10 were used to define a significant degree of heterogeneity [31, 32]. Fixed- and random-effects models were evaluated for the MA. Statistical significance was defined as *p* < 0.05. All analyses were conducted using the meta package in R software.

## Results

### Search and selection results

Of the 644 publications identified through searches made on electronic databases, 402 articles were excluded. The remaining 242 publications were assessed, of which 24 were eligible and included in the SLR (Fig. 1). Seventeen studies were RCTs, 4 were observational studies, and the remaining 3 were single-arm trials. The studies were performed in a broad range of countries, including the United States of America (USA) (7 studies), USA/Canada (3 studies), and Japan (13 studies), while 1 study was conducted globally.

During the feasibility assessment for the MA, 10 records were excluded. Finally, 14 studies were included for the MA (Fig. 1). All 14 studies were included in the safety analysis. Of the 14 studies, 8 were included in the efficacy analysis, while the remaining 6 were excluded either due to limited data (SD/SE/95% CI data absent) or no data at Week 1.

### Risk of bias

#### Randomized controlled trials

**Fig. 2:**
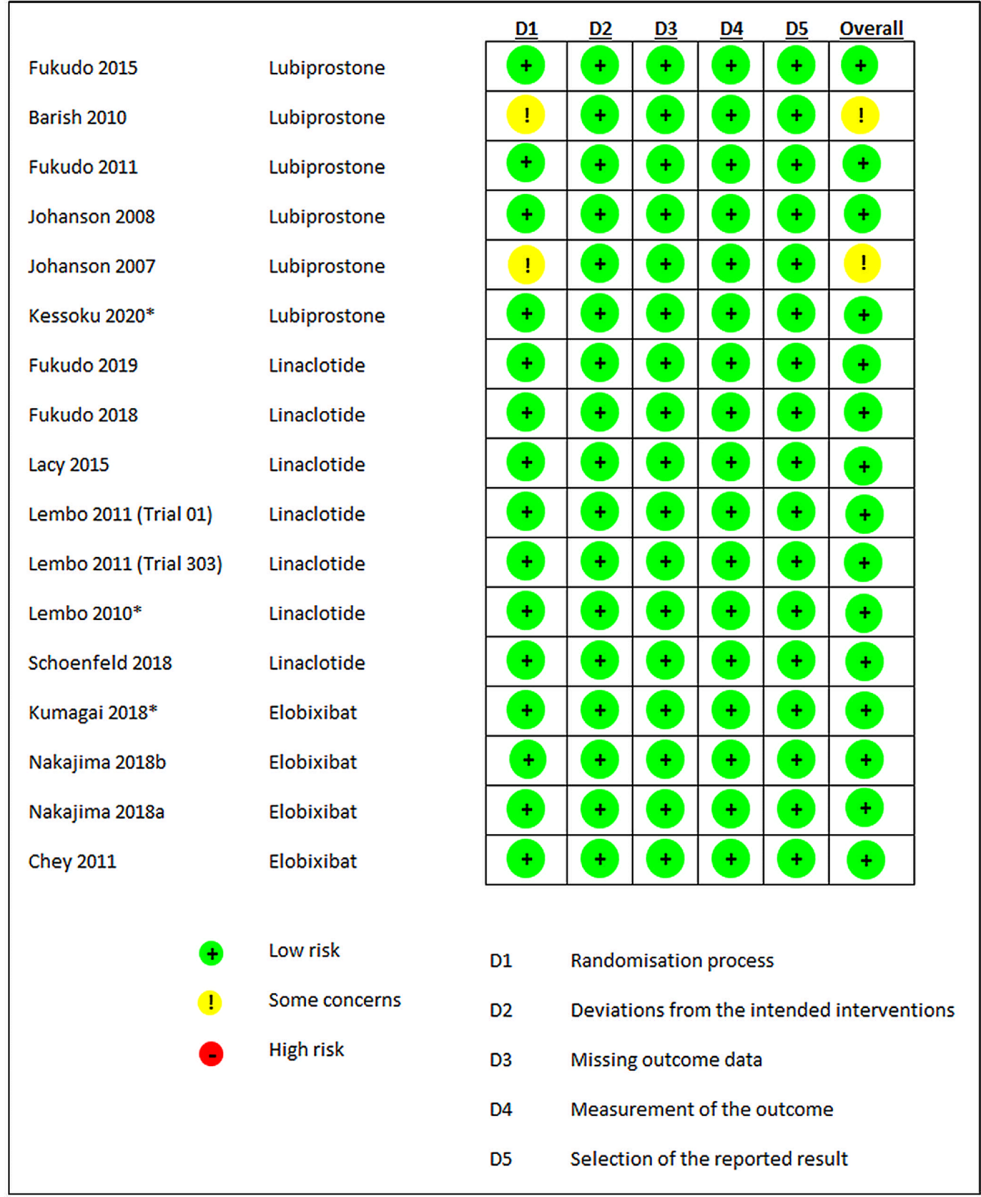
Risk of bias summary: judgments about each risk of bias item for each included study

The summary of the risk of bias of the RCTs for all products is presented in Fig. 2. Judgments about each risk of bias item presented as percentages for each intervention of interest are shown in supplementary Fig. S1.

#### Observational and single-arm trials

The NOS score was used to assess the quality of observational and single-arm studies. The quality of the included studies is detailed in supplementary Table S2.

### Efficacy

The efficacy data on SBM outcomes for all drugs are presented in Table 1.

**Table 1:** SBM outcomes for lubiprostone, linaclotide, and elobixibat

Author name, year	Drug	Study population and design	Endpoint	Results
Eguchi, 2020	Lubiprostone	• Population: ≥65 years (80%) • Population type: CC • Sample size: 1,338 • Design: Retrospective single-arm study • Region: Japan	• SBM frequency (no./wk)	Week 2 SBM frequency: mean (SD) • Before: 2.7 (2.7) • After: 5.3 (6.9); *p <* 0.01 **Note**: There were 34 (2.5%) patients who did not respond to the 2 weeks treatment with Lubi.
Fukudo, 2015	Lubiprostone	• Population: Adults • Population type: CIC • Sample size: 124 • Design: RCT, phase III • Region: Japan	• Change from baseline in SBM frequency (no./wk) • SBM ≤ 24 h • SBM ≤ 48 h • Responder rate (> 4 SBMs/wk)	SBM frequency, mean (SD) at baseline, • Lubi 48 mcg: 1.65 (0.78) • PBO: 1.68 (0.77), *p =* 0.873 Change from baseline in SBM frequency, mean (SE) • Week 1: Lubi 48 mcg: 3.7 (2.8); PBO: 1.3 (1.8), (*p <* 0.001) • Week 2: Lubi 48 mcg: 2.74; PBO: 1.33, (*p <* 0.001) • Week 3: Lubi 48 mcg: 2.75; PBO: 1.51, (*p <* 0.005) • Week 4: Lubi 48 mcg: 2.56; PBO: 1.62, (*p =* 0.042) SBM ≤ 24 h (%): • Lubi 48 mg: 58.1%; PBO: 30.6%, (*p =* 0.004) SBM ≤ 48 h (%): • Lubi 48 mcg: 80.6%; PBO: 64.5%, (*p =* 0.069) Responder rate (%) • Week 1: Lubi 48 mcg: 75.4%; PBO: 29.0% (*p =* 0.001) • Week 2: Lubi 48 mcg: 53.4%; PBO: 40.0% (*p =* 0.196) • Week 3: Lubi 48 mcg: 54.4%; PBO: 37.7% (*p =* 0.096) • Week 4: Lubi 48 mcg: 54.2%; PBO: 36.7% (*p =* 0.066)
Fukudo, 2011	Lubiprostone	• Population: Adults • Population type: CIC plus IBS-C • Sample size: 170 • Design: RCT phase II (dose-finding study) • Region: Japan	• Change from baseline in SBM frequency (no./wk) • SBM ≤ 24 h • SBM ≤ 48 h	Week 1 Change from baseline in SBM frequency, mean (SE) • Lubi 48 mcg: 6.8 (1.1), (*p* < 0.0001, vs. PBO) • PBO: 1.5 (0.4) SBM ≤ 24 h (n%) • Lubi 48 mcg: 75%, (*p <* 0.0001, vs. PBO) • PBO: 26.2% SBM ≤ 48 h (n%): • Lubi 48 mcg: 97.7%, (*p <* 0.0001, vs. PBO) • PBO: 57.1%
Johanson, 2008	Lubiprostone	• Population: Adults • Population type: CC • Sample size: 242 • Design: RCT; phase III • Region: USA	• SBM frequency (no./wk) • SBM ≤ 24 h • SBM ≤ 48 h • Responder rate (≥ 3 SBMs/wk)	SBM frequency, mean (SD) • Baseline: Lubi: 1.37 (0.87); PBO: 1.47 (1.33) • Week 1: Lubi: 5.69; PBO: 3.46 (*p* = 0.0001) • Week 2: Lubi: 5.06; PBO: 3.18 (*p* ≤ 0.002) • Week 3: Lubi: 5.25; PBO: 2.84 (*p* ≤ 0.002) • Week 4: Lubi: 5.30; PBO: 2.91 (*p* ≤ 0.002) SBM ≤ 24 h (%) • Lubi: 56.7%; PBO: 36.9%; (*p = ≤* 0.0024, vs. PBO) SBM ≤ 48 h (%) • Lubi: 80%; PBO: 60.7%; (*p* = 0.0013, vs. PBO) Responder rate (%) • Week 1: Lubi: 64.7%; PBO: 43.4% (*p <* 0.004) • Week 2: Lubi: 57.8%; PBO: 36.1% (*p <* 0.004) • Week 3: Lubi: 56.0%; PBO: 28.7% (*p <* 0.004) • Week 4: Lubi: 57.8%; PBO: 27.9% (*p <* 0.004)
Johanson, 2007	Lubiprostone	• Population: Adults • Population type: CC • Sample size: 127 • Design: RCT; phase II (dose-ranging study) • Region: USA	• SBM frequency (no./wk) • SBM ≤ 24 h	SBM frequency Week 1 • Lubi 48 mcg: (*p =* 0.002, vs. PBO) Week 2 • Lubi 48 mcg: (*p ≤* 0.020, vs. PBO) SBM ≤ 24 h (%) • Lubi 48 mcg (24 mcg b.d.): 59.4%; (*p =* 0.009)
Barish, 2010	Lubiprostone	•Population: Adults •Population type: CC •Sample size: 237 •Design: RCT; phase 3; global	• Frequency of SBMs (no./wk) • SBM ≤ 24 h (%) • Responder rate (%): • Full responders: (SBM frequency ≥ 4 per week)	Frequency of SBMs •Baseline: Lubi 48 mcg:1.28, PBO: 1.5 (*p* = 0.0126) •Week 1: Lubi 48 mcg: 5.89, PBO: 3.99 (*p* < 0.0001) •Week 2: Lubi 48 mcg: 4.96, PBO: 3.55 (*p* < 0.0487) •Week 3: Lubi 48 mcg: 5.56, PBO: 3.36 (*p* < 0.0004) •Week 4: Lubi 48 mcg: 5.37; PBO: 3.46 (*p* < 0.0068) SBM ≤ 24 h (%): •Week 1: Lubi 48 mcg: 61.3% •PBO: 31.4% (*p* < 0.0001) Responder rate, full responder (n%) •Week 1: Lubi 48 mcg: 72%, PBO: 49%; (*p* < 0.0001) •Week 2: Lubi 48 mcg: 58%, PBO: 43%; (*p* = 0.0171) •Week 3: Lubi 48 mcg: 61%, PBO: 36%; (*p* = 0.0002) •Week 4: Lubi 48 mcg: 60%, PBO: 39%; (*p* = 0.0002)
Fukudo, 2019	Linaclotide	• Population: Adults • Population type: CC • Sample size: 181 • Design: RCT, phase III • Region: Japan	• SBM frequency (no./wk) • Change from baseline in SBM frequency (no./wk) • SBM ≤ 24 h • Responder rate (> 3 SBMs/wk)	Week 1 SBM frequency: mean (95% CI) • Lina 500 mcg: 5.72 (5.10, 6.35) • PBO: 3.19 (2.55, 3.82); *p <* 0.001 Change from baseline in SBM frequency, mean (95% CI) • Lina 500 mcg: 4.02 (3.39, 4.64) • PBO: 1.48 (0.85, 2.12); *p <* 0.001 SBM ≤ 24 h (%, 95% CI) • Lina 500 mcg:72.8 (62.6, 81.6); *p <* 0.001 • PBO: 48.3 (37.6, 59.2) Responder rate: Mean (95% CI). 1st week: • Lina 500 mcg: 83.5 (74.3, 90.5) • PBO: 56.8 (45.8, 67.3); *p <* 0.001 2nd of 4 weeks: • Lina 500 mcg: 83.5 (74.3, 90.5) • PBO: 64.8 (53.9, 74.7); *p =* 0.006 3rd of 4 weeks: • Lina 500 mcg: 71.4 (61.0, 80.4) • PBO: 42.0 (31.6, 53.0); *p <* 0.001
Fukudo, 2018	Linaclotide	• Population: Adults • Population type: CC • Design: RCT; phase II (dose-finding study) • Sample size: 382 • Region: Japan	• SBM frequency (no./wk) • Change from baseline in SBM frequency (no./wk) • Responder rate (%) (> 3 SBMs/wk)	SBM frequency: mean. Week 1 • Lina 500 mcg: 5.58; *p <* 0.001 • PBO: 3.64 Week 2 • Lina 500 mcg: 5.70; *p <* 0.001 • PBO: 3.27 Change from baseline in SBM frequency, Mean. Week 1 • Lina 500 mcg: 3.85; *p <* 0.001 • PBO: 1.91 Week 2 • Lina 500 mcg: 3.96; *p <* 0.001 • PBO: 1.53 Responder Rate (%): Week 1 • Lina 500 mcg: 77.6%; *p =* 0.037 • PBO: 61.3% Week 2 • Lina 500 mcg: 82.7%; *p =* 0.002 • PBO: 60%
Schoenfeld, 2018	Linaclotide	• Population: Adults • Population type: CIC • Design: RCT; phase III, (NCT02291679) • Sample size: 1223 • Region: USA	• SBM frequency (no./wk) • Change from baseline in SBM frequency (no./wk)	Week 12 SBM frequency: mean. • Lina 145 mcg: 4.1 • PBO: 2.7 Change from baseline in SBM frequency, mean. • Lina 145 mcg: 2.6; *p <* 0.0001 • PBO: 1.3
Lacy, 2015	Linaclotide	• Population: Adults • Population type: CIC • Design: RCT; phase IIIb, (NCT01642914) • Sample size: 483 • Region: USA/Canada	• SBM frequency (no./wk) • Change from baseline in SBMs/wk • Change from baseline in days/wk with an SBM • SBM ≤ 24 h	Week 12 SBM frequency: mean • Lina 145 mcg: 5.2* • PBO: 3.3* *Note: Over the 12-week treatment period. Change from baseline in SBM frequency, LS mean • Lina 145 mcg: 3.6; (*p <* 0.0001, vs. PBO) • PBO: 1.6 Change from baseline in days/week with an SBM: mean • Lina 145 mcg: 2.3; (*p <* 0.0001, vs. PBO) • PBO: 1.2 SBM ≤ 24 h (%): • Lina 145 mcg: 61.4%; (*p <* 0.0006, vs. PBO)
Lembo, 2011a Lembo, 2011b	Linaclotide	• Population: Adults • Population type: CC • Design: RCT (Trial 303); phase III; (NCT00730015) • Sample Size: 642 • Region: USA/Canada	• SBM frequency (no./wk) • Change from baseline in SBM frequency (no./wk) • SBM ≤ 24 h • Increase of ≥ 2 SBMs for 9 of 12 wk	Week 12 SBM frequency: mean • Lina 145 mcg: 5.2 • PBO: 3.2 Change from baseline in SBM frequency, mean • Lina 145 mcg: 3.0; *p <* 0.001 • PBO: 1.1 SBM ≤ 24 h (%): • Lina 145 mcg: 70% *p <* 0.001 • PBO: 39.7% Increase of ≥ 2 SBMs for 9 of 12 wk (%) • Lina 145 mcg: 41% *p* < 0.001 • PBO: 12.9%
	Linaclotide	• Population: Adults • Population type: CC • Design: RCT (Trial 01); phase III; (NCT00765882) • Sample size: 630 • Region: USA/Canada	• SBM frequency (no./wk) • Change from baseline in SBM frequency (no./wk) • SBM ≤ 24 h • Increase of ≥ 2 SBMs for 9 of 12 wk	Week 12 SBM frequency: mean • Lina 145 mcg: 5.3 • PBO: 3.0 Change from baseline in SBM frequency, mean • Lina 145 mcg: 3.4; *p <* 0.001 • PBO: 1.1 SBM ≤ 24 h (%): • Lina 145 mcg: 64.3%; *p <* 0.001 • PBO: 39.1 Increase of ≥ 2 SBMs for 9 of 12 wk (%): • Lina 145 mcg: 39% P < 0.001 • PBO: 16.3
Tomie, 2020	Elobixibat	• Population: Elderly • Population type: CC • Design: RCS • Sample size: 104 • Region: Japan	• SBM frequency (no./wk) • SBM ≤ 24 h • Time to first SBM • Responder rate	Week 2 SBM frequency, mean (SD) • Baseline: 2.86 (1.77) • Elob: 6.08 (4.65); *p <* 0.001 Subgroup Age: ≤74 years: • Baseline: 2.82 (1.85) • Elob: 6.31 (4.77); *p <* 0.001 Age: ≥75 years: • Baseline: 2.90 (1.68) • Elob: 5.82 (4.54); *p <* 0.001 SBM ≤ 24 h (%): • Overall: 78.7% • Aged ≤ 74 years: 78.3%; *p* = 0.92 • Aged ≥ 75 years: 79.3%; *p* = 0.92 Time to first SBM, mean (SD) • Week 2: 14.8 (12.6) hrs Responder rate, (n%): • Week 2: 74%
Kumagai, 2018	Elobixibat	• Population: Adult • Population type: CC • Design: RCT, phase I (dose-escalating design) • Sample size: 120 • Region: Japan	• SBM ≤ 24 h	Week 2 SBM ≤ 24 h (%) • Elob 10 mg/day: 100% • Elob 15 mg/day: 88.9% • PBO: 40% The time to the first SBM was shorter in the Elob groups than in the PBO
Nakajima, 2018b	Elobixibat	• Population: Adult • Population type: CC plus IBS-C • Design: RCT, phase IIb (JapicCTI-142,608) • Sample size: 163 • Region: Japan	• Change from baseline in SBM frequency (no./wk) • SBM ≤ 24 h • Time to first SBM	SBM frequency, mean at baseline • 1.6 to 1.8 Week 1 Change from baseline in SBM frequency, mean • Elob 10 mg: 5.7; (*p <* 0.001, vs. PBO) • Elob 15 mg: 5.6; (*p <* 0.001, vs. PBO) • PBO: 2.6 SBM ≤ 24 h (%): • Elob 10 mg: 90%; (*p <* 0.001, vs. PBO) • Elob 15 mg: 93%; (*p <* 0.001, vs. PBO) • PBO: 48% Time to first SBM, mean • Elob 10 mg: 8.2 h • Elob 15 mg: 8.5 h • PBO: 36.2 h
Nakajima, 2018a	Elobixibat	• Population: Adult • Population type: CC plus IBS-C • Design: RCT, phase III, (JapicCTI-153,061) • Sample size: 132 • Region: Japan	• Change from baseline in SBM frequency (no./wk) • SBM ≤ 24 h • Responder rate (> 3 SBMs/wk)	Change from baseline in SBM frequency, LS mean (SE), [95% CI] Week 1 • Elob 10 mg: 6·4 (0·6) [5.3–7.6] • PBO: 1.7 (0.2) [1.2–2] • Difference: 4.7(0.6) [3.4–5.9]; *p <* 0.0001 Week 2 • Elob 10 mg: 5.0 (0.4) [4.2–5.8] • PBO: 1.8 (0.2) [1.3–2.2] • Difference: 3.2(0.5) [2.3–4.1]; *p <* 0.0001 SBM ≤ 24 h (n %) • Elob 10 mg: 86% • PBO: 41% • Difference: 44%; *p <* 0.0001 Responder rate (%) Week 1 • Elob 10 mg: 94% • PBO: 60% • Difference: 34% Week 2; *p* < 0.0001 • Elob 10 mg: 92% • PBO: 63% • Difference: 29%; *p* < 0.0001
Chey, 2011	Elobixibat	• Population: Adults • Population type: CIC • Design: RCT; phase IIb (NCT01007123) • Sample size: 190 • Region: USA	• Change from baseline in SBM frequency (no./wk) • SBM ≤ 24 h • Time to first SBM	Change from baseline in SBM frequency, LS mean (95% CI) Week 1 • Elob 10 mg: 4.0 (2.9–5.0); *p <* 0.002 • Elob 15 mg: 5.4 (4.4–6.4); *p <* 0.001 • PBO: 1.7 (0.7–2.8) SBM ≤ 24 h (%): • Elob 10 mg: 74%, (*p =* 0.012, vs. PBO) • Elob 15 mg: 75%, (*p =* 0.012, vs. PBO) • PBO: 45% Time to first SBM, mean • Elob 10 mg: 12 h (*p =* 0.033, vs. PBO) • Elob 15 mg: 07 h (*p =* 0.039, vs. PBO) • PBO: 27 h

Abbreviations: b.d.: Twice daily; CC: Chronic constipation; CIC: Chronic idiopathic constipation; Elob: Elobixibat; IBS-C: Irritable bowel syndrome with constipation; Lina: Linaclotide; LS: Least squared; Lubi: Lubiprostone; mcg: Microgram; mg: Milligram; PBO: Placebo; RCS: Retrospective cohort study; RCT: Randomized controlled trial; SBM: Spontaneous bowel movement; SD: Standard deviation; SE: Standard error; t.d.s: Three times daily; USA: United States of America; wk: Week

#### Lubiprostone:

A total of 6 studies were performed using lubiprostone. Three studies were from Japan, 2 were from the USA, and 1 was a global multicenter study. SBM data were reported for Weeks 1, 2, 3, 4, 12, and 48 in the included studies.

SBM frequency (no./wk): Overall, 3 studies reported this parameter. In the first RCT, lubiprostone induced a significantly greater mean SBM frequency at Week 1 versus placebo (5.69 vs. 3.46, *p* = 0.0001), and this effect was sustained over subsequent study weeks [33]. There was a significant change (*p <* 0.001) in baseline at Week 1 in SBM frequency (mean increase of 3.7 in lubiprostone group vs.1.3 in placebo group). Similar outcomes were observed at Week 2 (*p <* 0.001), Week 3 (*p <* 0.005), and Week 4 (*p* = 0.042) as placebo [34]. A phase II study also demonstrated a statistically significant, dose-dependent increase in change from baseline in the average weekly number of SBMs at Week 1 (mean [SE], placebo: 1.5 [0.4]; 32 mcg: 3.5 [0.5]; and 48 mcg: 6.8 [1.1] per week) [35]. In an observational study conducted among elderly patients, the mean SBM frequency increased significantly after Week 2 from 2.7 to 5.3 times/week (*p <* 0.01) [36].

SBM ≤ 24 h: Overall, 4 studies reported data for the endpoint. Lubiprostone showed a significant (*p* = 0.004) increase in the proportion of patients who achieved their first SBM within 24 h compared with those who received placebo (58.1% vs. 30.6%, respectively) [34]. A phase II study also reported a significant increase in the proportion of patients for SBMs within 24 and 48 h versus (SBM ≤ 24 h [75.0% vs. 26.2%, *p <* 0.0001] and SBM ≤ 48 h [97.7% vs. 57.1%, *p <* 0.0001], respectively) [35]. In a double-blind, multicenter study, a significantly higher percentage of patients experienced SBM within 24 h after the first dose of lubiprostone (56.7% vs. 36.9%, respectively, *p* ≤ 0.0024), as well as within the first 48 h of the dose (80.0% vs. 60.7%, respectively, *p =* 0.0013) [33]. Similarly, significant improvement was observed within 24 h of treatment with lubiprostone 48 mcg versus placebo (61.3% vs. 31.4%; *p <* 0.0001) [37].

Responder rate: It was reported in 3 studies. The response rate (SBM ≥ 4 per week) was significantly higher in the lubiprostone group versus the placebo group (75.4% vs. 29.0%, respectively; *p =* 0.001). However, no significant difference was observed in subsequent weeks for lubiprostone versus placebo [34]. The percentage of full responders (SBM ≥ 3 per week) was significantly higher compared to placebo for each treatment week up to 4 weeks (*p <* 0.004) [33]. Similarly, a multicenter study reported a significantly higher response rate (SBM ≥ 4 per week) with lubiprostone compared to placebo at Weeks 3 and 4 (*p =* 0.0002) [37].

#### Linaclotide:

Six studies reported data for linaclotide, of which 2 each from Japan, the USA, and USA/Canada were included. Sample sizes ranged from 181 to 1223. The treatment durations were 2, 4, and 12 weeks.

SBM frequency (no./wk): Overall, 5 studies reported this parameter. At Week 1, a significantly greater mean SBM frequency was reported with linaclotide versus placebo (linaclotide 500 mcg: 5.72 vs. placebo: 3.19 times per week, *p* < 0.001) [38]. In another study at Week 2, linaclotide 500 mcg groups reportedly had a significant (*p <* 0.001) increase in the mean SBM frequency compared to placebo groups (5.70 vs. 3.27) [39]. Two USA/Canada-based trials also demonstrated a high mean SBM number per week at Week 12 (*p*-value not reported) [40]. In 2 phase III trials, at Week 12, the mean SBM frequency per week was reportedly higher in the linaclotide group compared to the placebo group; however, the *p*-value was not reported [41]. In CC patients, there was a significant change (*p <* 0.001) in the mean SBM frequency from baseline with linaclotide 500 mcg (4.02) compared to placebo (1.48) at Week 1 [38]. Another study from the USA/Canada also reported a significant change (*p <* 0.001) in SBM frequency from baseline compared to placebo (*p <* 0.001). There was a significant change in SBM frequency per week from baseline in patients treated with different linaclotide doses versus placebo at Week 12 (linaclotide 145 mcg: 2.6 vs. placebo: 1.3, *p <* 0.0001) [42]. Similarly, treatment with linaclotide 145 mcg or 290 mcg showed a significant improvement in SBM frequency per week from baseline at Week 12 (*p* < 0.0001) [41].

SBM ≤ 24 h: Overall, 3 studies reported this parameter. The linaclotide group reported a significantly (*p <* 0.001) higher percentage of patients experiencing SBM within 24 h after the first dose of linaclotide versus the placebo group (72.8% vs. 48.3%) [38]. A USA/Canada-based study showed a significantly higher percentage of SBM within 24 h after the initial dose of linaclotide 145 mcg at Week 12 (*p <* 0.001) versus placebo [40]. A significantly greater number of patients had the first SBM within 24 h after administration of the initial dose (*p <* 0.0006 with linaclotide 145 mcg and *p <* 0.0022 with linaclotide 290 mcg) [41].

Responder rate: It was reported in 3 studies. The percentage of responders (≥ 3 SBMs per week with an increase of ≥ 1 SBMs from baseline) was significantly higher with linaclotide versus placebo at Week 1 (83.5% vs. 56.8%; *p <* 0.001) and in subsequent weeks [38]. At Week 12, there was also a significantly (*p <* 0.001) high number of responders in the treatment group (increase of ≥ 2 SBMs for 9 of 12 weeks) compared to the placebo group [40].

#### Elobixibat:

Five studies reported data for elobixibat, of which 4 were from Japan and 1 was from the USA. Sample sizes ranged from 104 to 190. The treatment duration ranged from 1 to 8 weeks.

SBM frequency (no./wk): Only 1 observational study provided data for the mean SBM frequency after 2 weeks of administration in elderly patients (10 mg); the mean SBM frequency significantly increased from 2.86 to 6.08 times/week (*p <* 0.001) at Week 2, with the subgroup aged ≤ 74 seeing an increase from 2.82 to 6.31 and the subgroup aged ≥ 75 seeing an increase from 2.90 to 5.82 [43]. There was a highly significant change in SBM frequency from baseline with elobixibat (10 mg and 15 mg) at Weeks 1 and 2 compared to placebo (*p <* 0.0001) [44]. In another study, a significant change from baseline was observed with elobixibat 10- and 15-mg doses (*p <* 0.001) versus placebo at Weeks 1 and 2 [45]. Patients treated with 10 mg or 15 mg of elobixibat showed a significantly (*p <* 0.002 and *p <* 0.001, respectively) greater change versus placebo in the number of SBMs (10 mg: 74%; 15 mg: 75% vs. PBO: 45%) at Week 1 [46].

SBM ≤ 24 h: Three studies reported a significant increase in the number of patients having SBM within 24 h after the first dose of elobixibat (10 mg) in the CC plus IBS-C (86–90%) and CIC (74%) populations, respectively [44–46].

Responder rate: It was reported in 1 of the studies conducted among CC plus IBS-C patients. The percentage of responders (SBM ≥ 3 per week) was significantly higher with elobixibat 10 mg compared with placebo at Week 1 (94%) and Week 2 (92%) (*p*-value not reported) [45].

### Patient symptoms

The summarized data for patient symptoms are presented as “stool consistency and straining severity” in supplementary Table S3, “abdominal bloating and abdominal pain” in supplementary Table S4, and “constipation severity” in supplementary Table S5. Lubiprostone significantly improved stool consistency, straining severity, and constipation severity in every study; abdominal bloating in 2 of 3 studies; and abdominal pain/discomfort in 1 of 3 studies. Linaclotide significantly improved stool consistency, straining severity, abdominal pain/discomfort, and constipation severity in every study and abdominal bloating in most of the studies. Elobixibat 5 mg and 10 mg significantly improved stool consistency in every study, straining severity (5 mg and 10 mg), abdominal bloating (15 mg), and constipation severity (10 mg) in only one study that observed each symptom, but not abdominal pain/discomfort (10 mg) in one study.

### Safety outcomes

A total of 21 studies reported safety data, with 9 studies for lubiprostone and 6 each for linaclotide and elobixibat. Treatment-emergent adverse events (TEAEs) observed across the included studies were diarrhea, nausea, abdominal pain, vomiting, nasopharyngitis, and upper respiratory tract infection. Diarrhea was the most frequent AE reported in most of the studies. Overall, the TEAEs reported in different studies are presented in supplementary Table S6.

### QoL outcomes

#### Lubiprostone:

Three studies reported data for HRQoL [34, 35]. In Japan, a significant improvement in the SF-36 subscale (physical function, bodily pain, general health, vitality, and role emotional subscale) was observed at Weeks 24 and 42 [34]. Significant changes were also observed in IBS-QoL, with all the subscales improving at Weeks 24 and 48 in the study [34].

#### Linaclotide:

Four studies reported HRQoL data [38, 39, 42, 47]. Significant changes were observed in 2 trials (*p ≤* 0.001) in overall and subscale scores (physical discomfort, psychosocial discomfort, worries and concerns, and satisfaction) with linaclotide 145 mcg compared to placebo at Week 12 [39]. Another study reported significant improvement from baseline in overall PAC-QoL (*p ≤* 0.05) and subscale scores, as well as physical discomfort (*p ≤* 0.05) and satisfaction (*p ≤* 0.01), for all linaclotide doses (72 or 145 mcg) at Week 4 [42]. A significant change was not observed in the total IBS-QoL score at Week 4, but the total score and all the subscales were improved with linaclotide 500 mcg at Weeks 24 and 56 in studies from Japan [38].

#### Elobixibat:

A Japan-based study reported a significant improvement in the overall JPAC-QoL score and subscales (physical discomfort, psychosocial discomfort, worries and concerns, and satisfaction) with elobixibat (5, 10, 15 mg) from baseline at Weeks 4, 12, 24, 36, and 52 (p < 0.0001) in the CC population [45].

### Meta-analysis outcomes

#### SBM frequency at Week 1

**Fig. 3:**
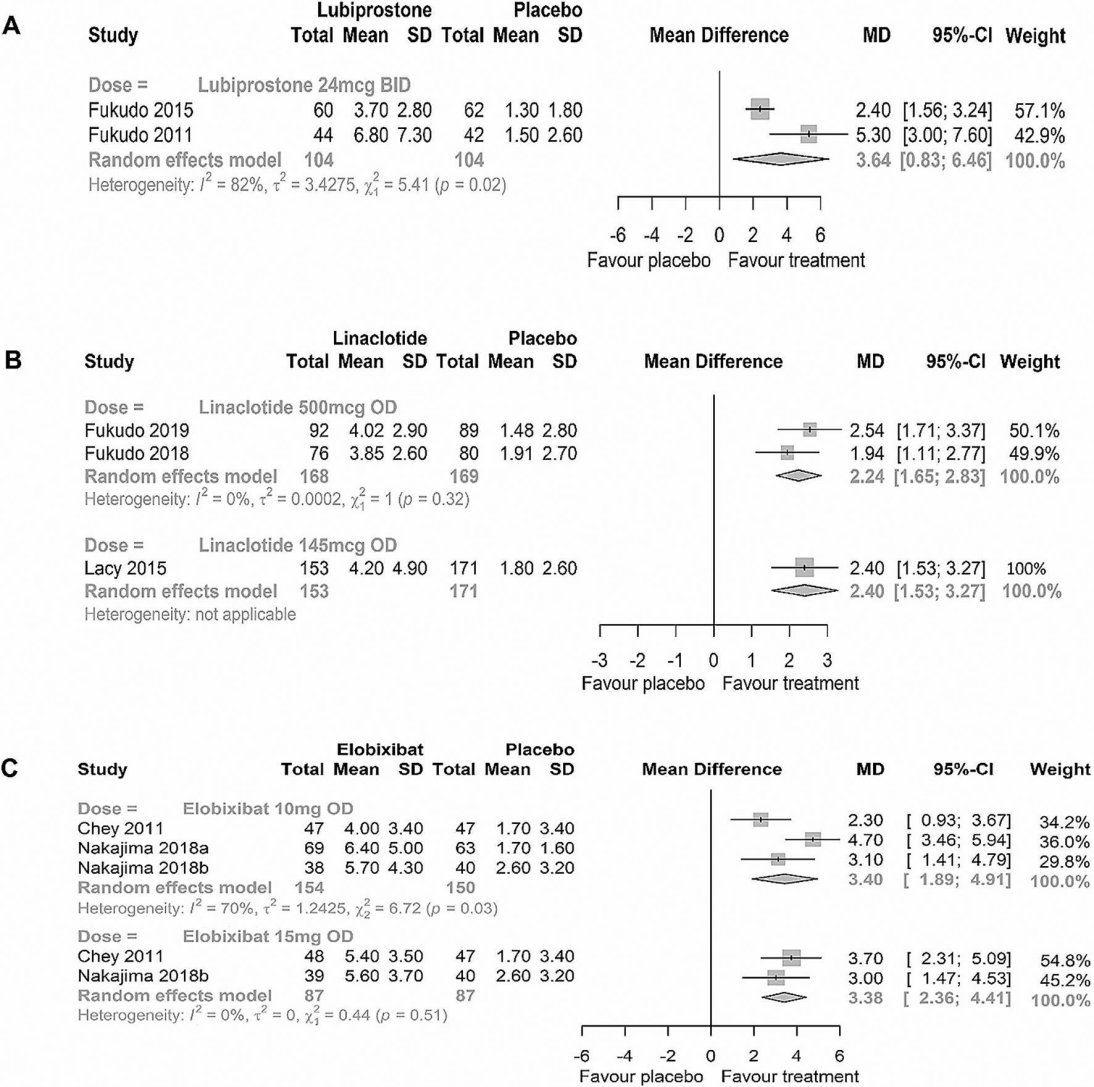
Forest plots for SBM frequency at Week 1 for A: lubiprostone, B: linaclotide, and C: elobixibat

The forest plots for SBM frequency at Week 1 are presented in Fig. 3.

*Lubiprostone*: Two studies were included [34, 35]. The pooled effect estimates showed that the mean change from baseline in SBM frequency was significantly higher with lubiprostone (MD: 3.64 [95% CI: 0.83–6.46], *p* = 0.0111) versus placebo; however, substantial heterogeneity existed in the analysis (*p* = 0.02, I^2^ = 82%).

*Linaclotide*: Three studies were included: 2 for linaclotide 500 mcg [38, 39] and 1 for linaclotide 145 mcg [41]. Linaclotide 500 mcg significantly improved the mean change from baseline in SBM frequency (MD: 2.24 [95% CI: 1.65–2.83], *p* < 0.0001) versus placebo, with no significant heterogeneity (*p* = 0.32, I^2^ = 0%). Similar results were reported for linaclotide 145 mcg (MD: 2.40 [95% CI: 1.53–3.27], *p* < 0.0001).

*Elobixibat*: A total 3 studies were included: 2 studies [44, 46] assessed the 2 different doses of elobixibat 10 mg and 15 mg, and the remaining study evaluated only the 10-mg dose [45]. The mean change from baseline in the frequency of SBM was significantly higher with elobixibat 10 mg (MD: 3.40 [95% CI: 1.89–4.91], p < 0.0001) and 15 mg (MD: 3.38 [95% CI: 2.36–4.41], p < 0.0001) than placebo, with significant heterogeneity (*p* = 0.03, I^2^ = 70%) noted between the 2 studies using 10 mg, and no significant heterogeneity (*p* = 0.51, I^2^ = 0%) noted in the study using 15 mg.

#### Proportion of patients with diarrhea

The forest plots for diarrhea are presented in Supplementary Fig. S2.

*Lubiprostone*: A total of 5 studies were included [13, 33–35, 37]. The RR for diarrhea was significantly higher for lubiprostone (RR 6.20 [95% CI: 2.14–17.99], *p* = 0.0008) versus placebo, with no significant heterogeneity (*p* = 0.72, I^2^ = 0%) noted between studies.

*Linaclotide*: Out of the 6 studies included, 4 assessed 145 mcg [40–42] and the other 2 evaluated 500 mcg [38, 39]. The RR for diarrhea was significantly higher with linaclotide 145 mcg (RR 3.13 [95% CI: 1.88–5.21]; *p* < 0.0001]) and 500 mcg (RR 10.11 [95% CI: 1.91–53.50]; *p* = 0.0065) than with placebo. There was no significant heterogeneity for 500 mcg (*p* = 0.80, I^2^ = 0%), but the results were contrary for 145 mcg (*p* = 0.09, I^2^ = 55%).

*Elobixibat*: A total of 3 studies were included for elobixibat: 2 studies [46] assessed the 2 different doses of 10 mg and 15 mg and the other evaluated only the 10 mg dose [45, 46]. Three studies with 10 mg and 2 studies with 15 mg were included in the MA, respectively [44, 46]. The pooled effect estimates revealed that both doses had a significantly greater risk of diarrhea, with an effect estimate of RR 5.62 (95% CI: 1.24–25.49; *p* = 0.0251) for 10 mg and RR 6.09 (95% CI: 1.12–33.19; *p* = 0.0367) for 15 mg. There was no significant heterogeneity between both doses (10 mg: *p* = 0.59, I^2^ = 0%; 15 mg: *p* = 0.93, I^2^ = 0%).

#### Sensitivity analysis

A sensitivity analysis was performed to verify the robustness of the results. Sensitivity analysis was carried out for the sample size. The analysis showed that the exclusion of studies with low and high sample sizes did not influence the pooled estimates for lubiprostone and linaclotide 145 mcg, respectively. The results of the sensitivity analysis are presented in Supplementary Figs. S3 and S4.

#### NNT and NNH

**Fig. 4:**
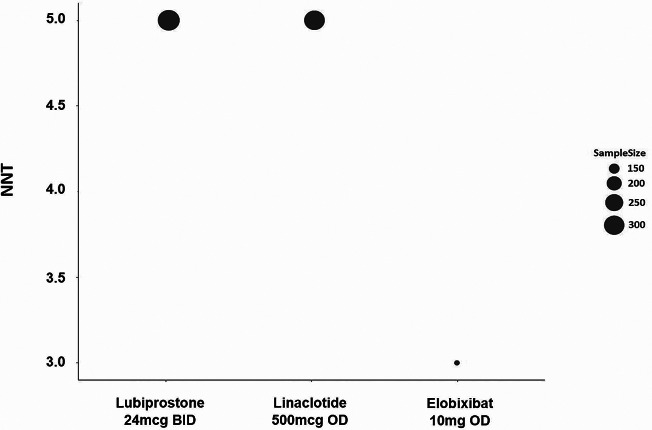
NNT values for the SBM frequency responder rate at Week 1

The NNT for the full responder rate at Weeks 1 was 3 (full responder defined as SBM frequency of ≥ 4 per week) and 5 (full responder defined as SBM frequency of ≥ 3 per week) for lubiprostone 24 mcg BID, respectively. The NNT for responder rate at Week 1 (defined as ≥ 3 SBM per week and an increase of at least one spontaneous bowel movement) was 5 for linaclotide 500 mcg and 3 for elobixibat 10 mg, respectively (Fig. 4).

**Fig. 5:**
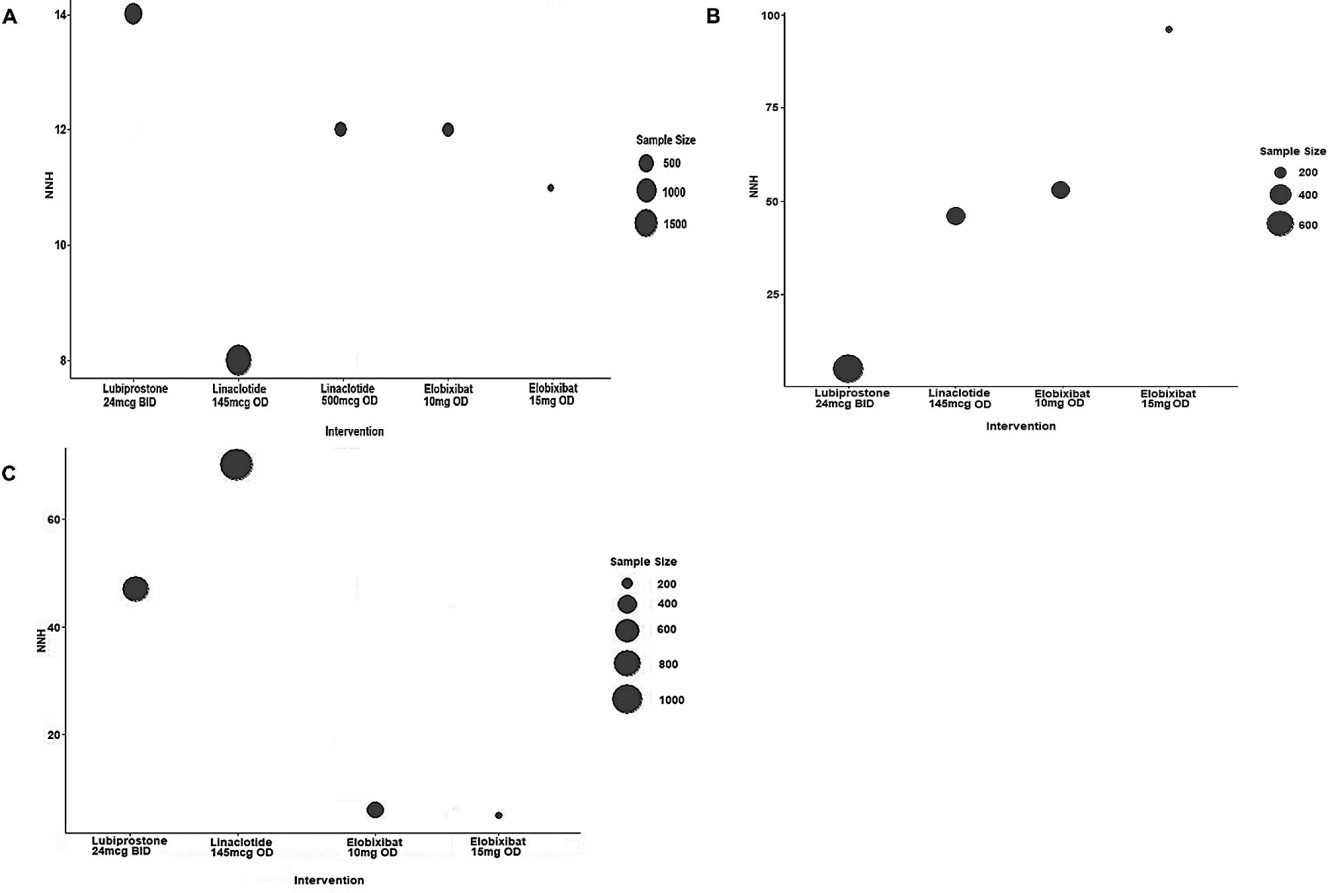
NNH values for A: diarrhea, B: nausea, and C: abdominal pain

The NNH for diarrhea was higher for lubiprostone (14, 95% CI: 10–23), followed by linaclotide 500 mcg (12, 95% CI: 8–26), elobixibat 10 mg (12, 95% CI: 8–27), elobixibat 15 mg (11, 95% CI: 6–44), and linaclotide 145 mg (8, 95% CI: 7–11). For nausea, elobixibat 15 mg was the highest (96, 95% CI: -26 to 17), followed by elobixibat 10 mg (53, 95% CI: -69 to 19), and, subsequently, linaclotide 145 mg (46, 95% CI: -69 to 17) and lubiprostone (5, 95% CI: 4 to 6). For abdominal pain, it was higher for linaclotide 145 mcg (70, 95% CI: -171 to 29), followed by lubiprostone (47, 95% CI: -209 to 21) and elobixibat 10 mg (6, 95% CI: 4 to 9) and 15 mg (5, 95% CI: 4 to 8) (Fig. 5).

Given the small sample sizes, NNT and NNH results must be interpreted with caution. Furthermore, NNT (for responder rate at Week 1) for linaclotide 145 mcg and elobixibat 15 mg and NNH (for nausea and abdominal pain) for linaclotide 500 mcg were not estimated due to no single study reporting the data.

## Discussion

Our systematic review compared the efficacy of three agents with SBM outcomes as primary outcome measures, which were dictated by the Rome committee during the period when these clinical trials were conducted. A significant improvement in SBM frequency at Week 1 was observed in the MA with treatment using approved doses of lubiprostone, linaclotide, or elobixibat.

The efficacy analysis revealed that mean SBM frequency significantly increased after 1 week of lubiprostone administration, both among adults and elderly patients. In the current study, lubiprostone significantly improved SBM frequency versus placebo (MD: 3.64); however, the results must be interpreted with caution due to the substantial heterogeneity (I^2^ = 82%) between trails. An MA conducted by Passos et al. also reported similar results, although it was conducted for different outcomes, such as the weekly responder rate and the SBM rate within 24 h [48]. The most common AE associated with lubiprostone was nausea, followed by abdominal bloating, headache, abdominal pain, diarrhea, and vomiting. From the SLR, nausea was noted to be the most common AE with lubiprostone across all geographies in the CC and CIC populations.

Linaclotide was approved at different doses: 500 mcg in Japan and 145 mcg in the USA or Canada, respectively, based on phase IIb studies conducted in each population [38]. The SLR showed a significantly greater mean SBM frequency compared to placebo among patients receiving linaclotide 500 mcg at Weeks 1 and 2. In the MA, both doses of linaclotide were found to significantly improve SBM frequency at Week 1 versus placebo. Comparable results were reported by Videlock et al., as linaclotide significantly improved the weekly complete spontaneous bowel movement (CSBM) responder rate [49]. The most common AE associated with linaclotide was diarrhea, with geographical differences observed regarding severity.

Elobixibat significantly improved SBM frequency and stool consistency in elderly CC patients. Elobixibat is not currently approved by the FDA but is available in Japan, where it was approved based on the results of 2 phase III trials. In the current study, diarrhea and abdominal pain were the most common AEs associated with elobixibat [45].

Considering the combined results of summarized outcomes (Table 1), MA (Fig. 3), and NNT (Fig. 4), no major differences in efficacy regarding SBMs were observed among the three drugs. However, the results should be interpreted with caution because the present study had no data for subgroup analysis. The efficacy of each drug might differ depending on patient characteristics. Additionally, long-term efficacy may also vary between the drugs since most of studies included in the current study were of relatively short durations. Therefore, physicians must consider the individual patient’s symptoms and backgrounds before prescribing interventions. Patient symptoms have been reported as secondary endpoints in most studies. Lubiprostone and linaclotide significantly ameliorated almost all the symptoms defined in the current study. These two drugs appear to have comparable effects on symptoms. Due to limited data of elobixibat evaluating straining severity, abdominal bloating, abdominal pain/discomfort, and constipation severity, it is not yet possible to adequately determine the effects of elobixibat.

QoL improvements were also observed with all 3 drugs with long-term treatment. Lubiprostone improved QoL both in Japan- and USA/Canada-based long-term studies (> 12 weeks). Linaclotide showed improved QoL across the USA and USA/Canada at Weeks 4 and 12. Only 1 study reported QoL data for elobixibat and showed a significant improvement in the Japanese CC population up to 52 weeks. Considering the treatment objective for CC, it was desirable to demonstrate the differences between these drugs in this study. However, the assessment tools used varied among clinical trials, such as SF-36 and IBS-QoL for lubiprostone, PAC-QoL and IBS-QoL for linaclotide, and JPAC-QoL for elobixibat. Therefore, further research is required to compare differences across the agents using the same assessment tools and treatment durations.

Interestingly, the current study indicated differences in the safety profiles of the three drugs, although the efficacy in SBM outcomes, effects on patient symptoms, and QoL improvement were relatively similar. Notably, the NNH analysis clearly indicated differences among them, as it was the lowest with lubiprostone for nausea, linaclotide for diarrhea, and elobixibat for abdominal pain, respectively. Given that side effects and physiological backgrounds vary across patients with CC/CIC, we think this may serve as a guide for doctors in choosing the right drug for each. [5]. Lubiprostone, which is prone to causing nausea, should be given along with instructions to avoid administration on an empty stomach. Given that the results from the MA for diarrhea (Fig. S2) also showed a higher risk ratio, linaclotide should be prescribed with caution, especially to patients who have already experienced diarrhea with other drugs. Elobixibat may not be an option for patients who complain of symptoms such as abdominal pain or discomfort.

The strength of this study is providing comprehensive and comparative information, not only about efficacy but also about the safety, patient symptoms, and QoL associated with these drugs. Although efficacy, defined as CSBM among laxatives, was presented in an earlier SLR and network MA [50], differences in safety were first elaborated by the NNH analysis in the current study. The characteristics of patients with constipation vary in terms of biological background, annoying symptoms, and other associated factors. Therefore, a personalized approach based on individual symptoms and comorbidities should be preferred for treatment. As for pharmacotherapy, safety is one of the most relevant factors because CC/CIC needs long-term treatment, and AEs can be a major cause of drug withdrawal.

This study has a few limitations. Firstly, there might have been a geographical bias because most of the studies were from Japan, followed by the USA, and one was a global multicenter study. Secondly, with respect to the MA, the total number of included studies was small and the treatment duration is only a week. Therefore, the current study did not assess the differences on long-term effectiveness among the agents. Thirdly and finally, we assessed efficacy using SBM, although CSBM is now a frequently reported primary outcome, because there were few studies that reported CSBM outcomes and none for lubiprostone among the trials included in the current study. Therefore, future research with more studies is needed.

In conclusion, the current study provides an updated overview of the efficacy, safety, patient symptoms, and QoL of 3 new drugs (viz.lubiprostone, linaclotide, and elobixibat) with different mechanisms of action for CC. Our findings may help physicians adopt an individualized approach to treating CC in clinical practice. Further studies are required to detect the appropriate population for each drug to address the other unmet needs of patients with CC.

### Electronic supplementary material

Below is the link to the electronic supplementary material.

**Supplementary Material 1: Figure S1.** Risk of bias graph: judgments about each risk of bias item presented as percentages for A: lubiprostone, B: linaclotide, and C: elobixibat; **Figure S2.** Forest plots for diarrhea for A: lubiprostone, B: linaclotide, and C: elobixibat​; **Figure S3.** Forest plots of sensitivity analysis by using fixed and random-effects model according to intervention for proportion of patients with diarrhea. A: lubiprostone 48 mcg fixed effect, B: lubiprostone 48 mcg random effect; **Figure S4.** Forest plots of sensitivity analysis by using fixed and random-effects model according to intervention for proportion of patients with diarrhea. A: linaclotide 145 mcg fixed effect, B: linaclotide 145 mcg random effect

**Supplementary Material 2: S1 Table.** PICOS criteria for SLR; **S2 Table.** NOS for assessing the quality of observational and single arm trial studies; **S3 Table.** Stool consistency and straining severity for lubiprostone, linaclotide, and elobixibat; **S4 Table.** Abdominal bloating, pain/discomfort for lubiprostone, linaclotide, and elobixibat; **S5 Table.** Constipation severity for lubiprostone, linaclotide, and elobixibat; **S6 Table.** Safety outcomes for lubiprostone, linaclotide, and elobixibat

## Data Availability

All data generated or analyzed during this study are included in this published article and its supplementary information files.
