# Supplementary material for: Comparative profiles of lubiprostone, linaclotide, and elobixibat for chronic constipation: a systematic literature review with meta-analysis and number needed to treat/harm
Source: BMC Gastroenterol. 2024 Jan 2;24:12. doi: 10.1186/s12876-023-03104-8 (PMC10759335; doi:10.1186/s12876-023-03104-8)
Supplement: Supplementary file 1 — Supplementary Material 1: Figure S1. Risk of bias graph: judgments about each risk of bias item presented as percentages for A: lubiprostone, B: linaclotide, and C: elobixibat; Figure S2. Forest plots for diarrhea for A: lubiprostone, B: linaclotide, and C: elobixibat​; Figure S3. Forest plots of sensitivity analysis by using fixed and random-effects model according to intervention for proportion of patients with diarrhea. A: lubiprostone 48 mcg fixed effect, B: lubiprostone 48 mcg random effect; Figure S4. Forest plots of sensitivity analysis by using fixed and random-effects model according to intervention for proportion of patients with diarrhea. A: linaclotide 145 mcg fixed effect, B: linaclotide 145 mcg random effect [file 12876_2023_3104_MOESM1_ESM.pptx]

## Slide 1
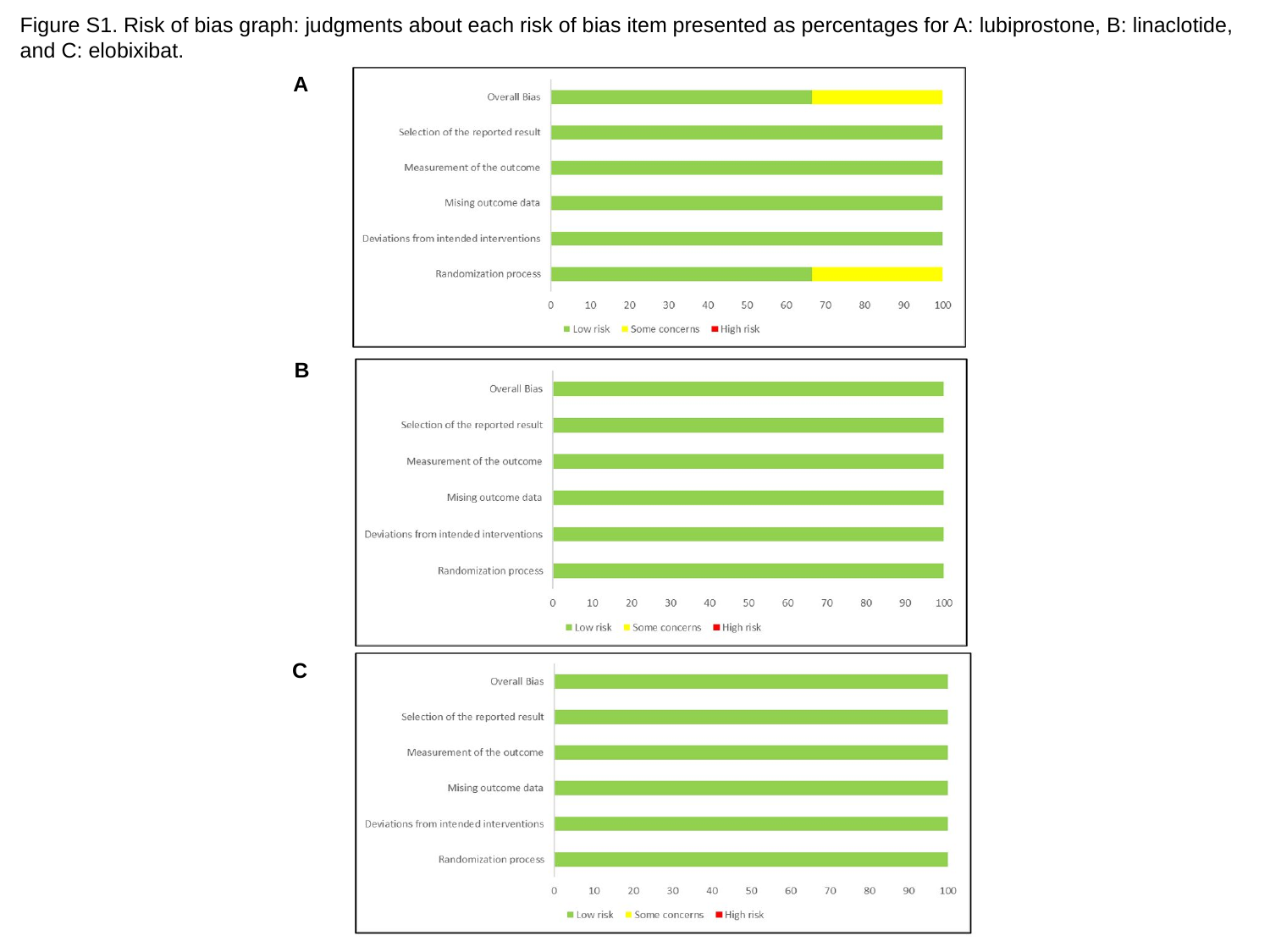

Figure S1. Risk of bias graph: judgments about each risk of bias item presented as percentages for A: lubiprostone, B: linaclotide, and C: elobixibat.
A
B
C

## Slide 2
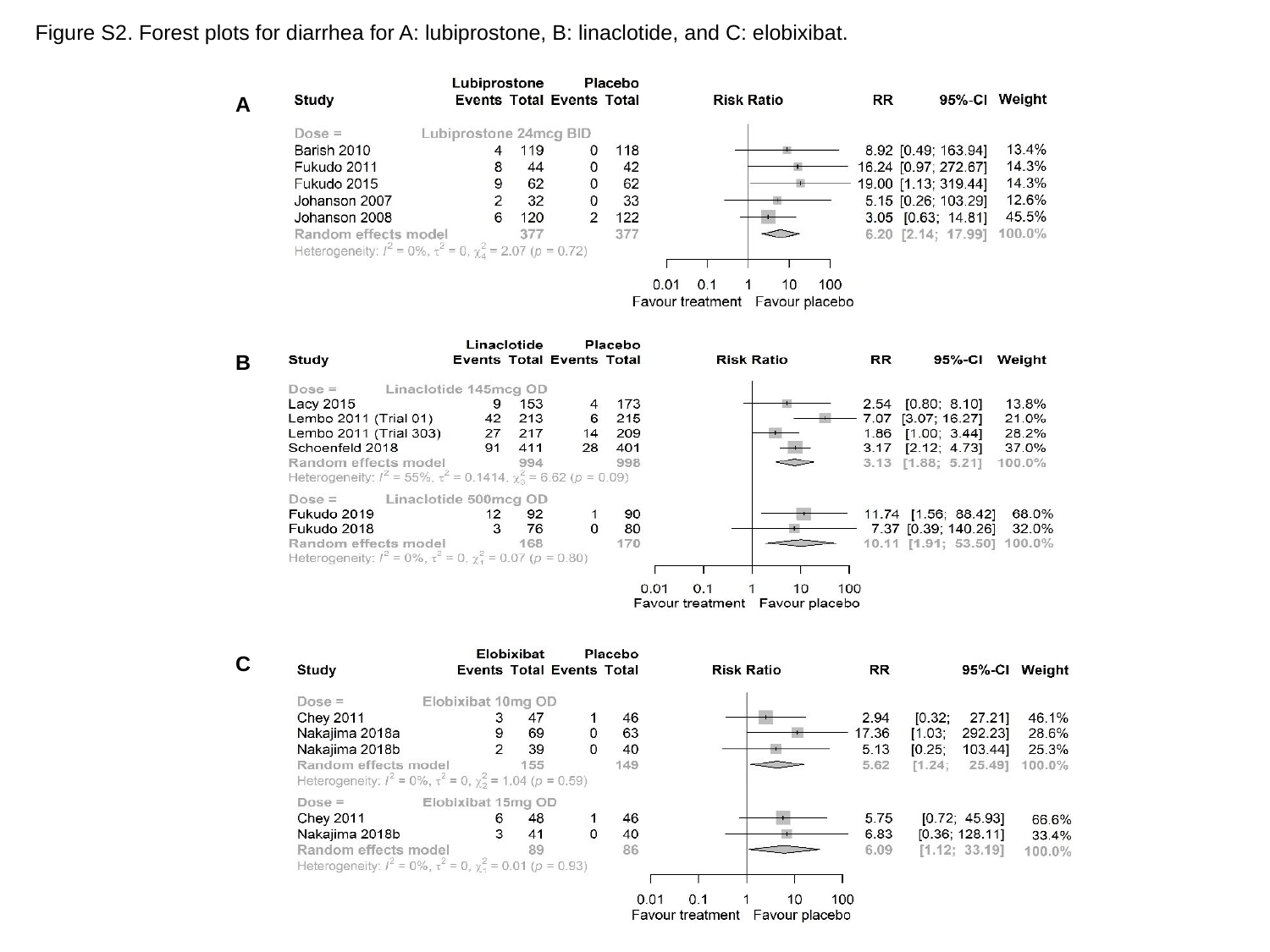

Figure S2. Forest plots for diarrhea for A: lubiprostone, B: linaclotide, and C: elobixibat.
A
B
C

## Slide 3
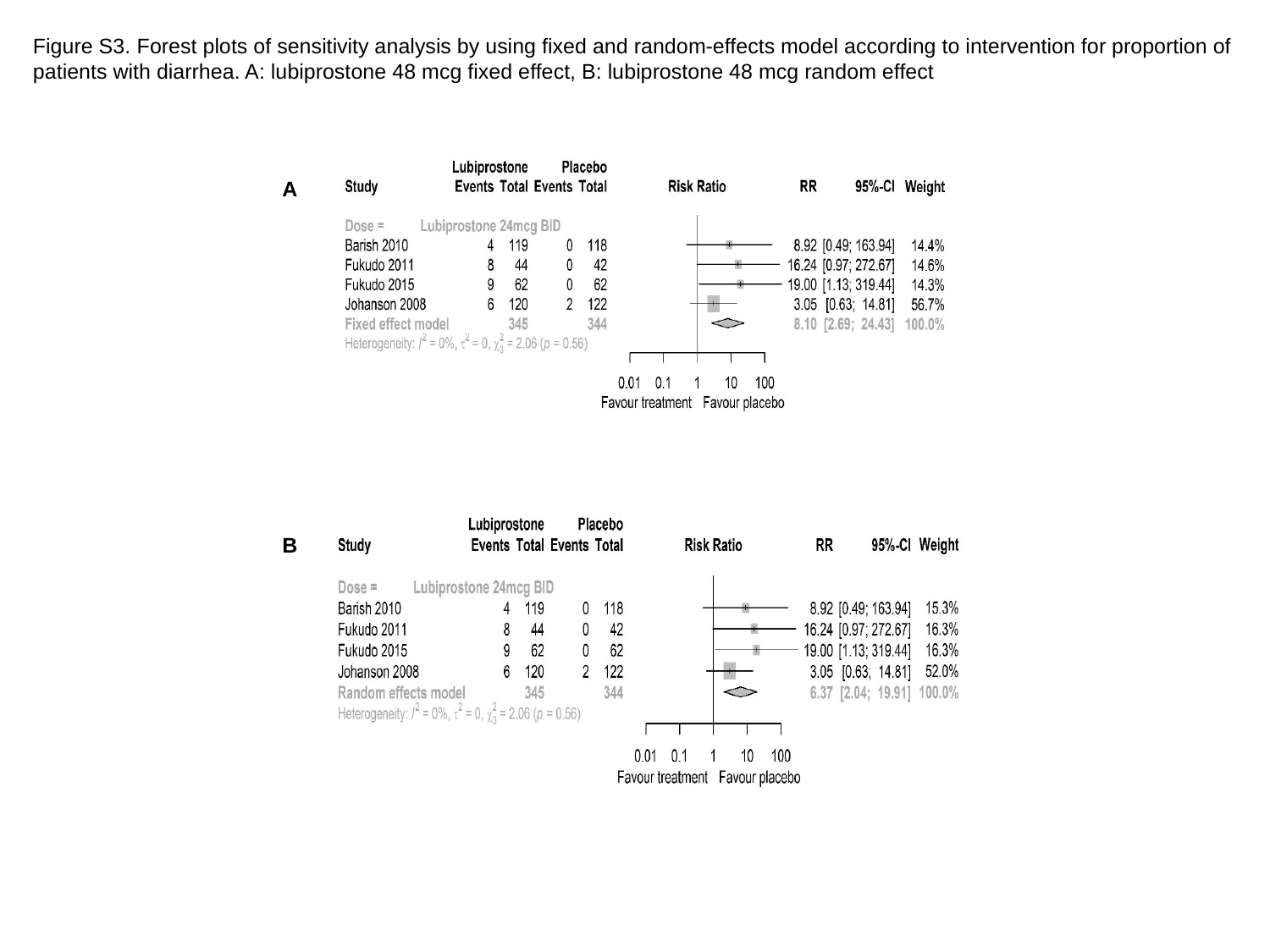

Figure S3. Forest plots of sensitivity analysis by using fixed and random-effects model according to intervention for proportion of patients with diarrhea. A: lubiprostone 48 mcg fixed effect, B: lubiprostone 48 mcg random effect
A
B

## Slide 4
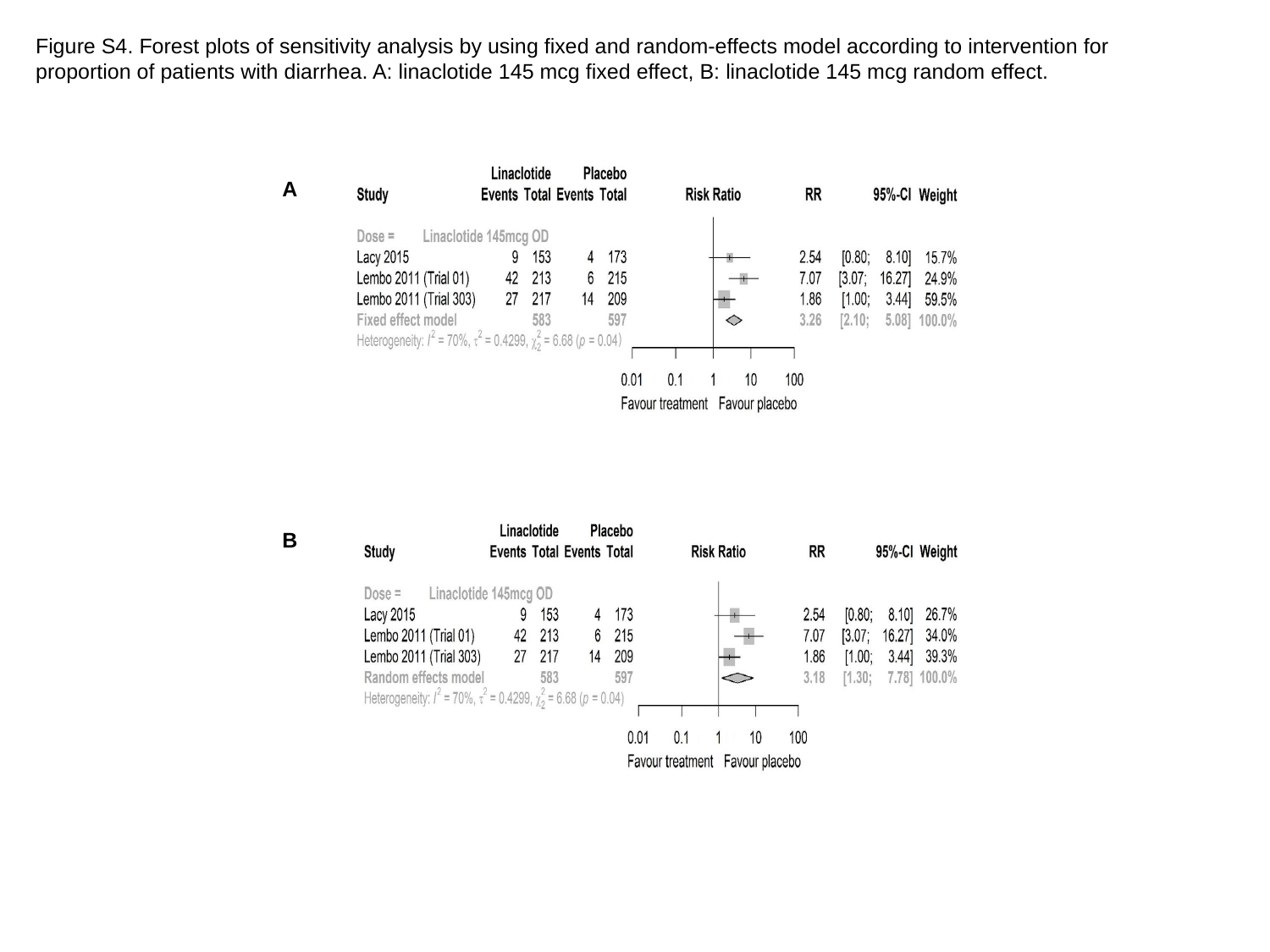

Figure S4. Forest plots of sensitivity analysis by using fixed and random-effects model according to intervention for proportion of patients with diarrhea. A: linaclotide 145 mcg fixed effect, B: linaclotide 145 mcg random effect.
A
B
