# Supplementary material for: Comparative profiles of lubiprostone, linaclotide, and elobixibat for chronic constipation: a systematic literature review with meta-analysis and number needed to treat/harm
Source: BMC Gastroenterol. 2024 Jan 2;24:12. doi: 10.1186/s12876-023-03104-8 (PMC10759335; doi:10.1186/s12876-023-03104-8)
Supplement: Supplementary file 2 — Supplementary Material 2: S1 Table. PICOS criteria for SLR; S2 Table. NOS for assessing the quality of observational and single arm trial studies; S3 Table. Stool consistency and straining severity for lubiprostone, linaclotide, and elobixibat; S4 Table. Abdominal bloating, pain/discomfort for lubiprostone, linaclotide, and elobixibat; S5 Table. Constipation severity for lubiprostone, linaclotide, and elobixibat; S6 Table. Safety outcomes for lubiprostone, linaclotide, and elobixibat [file 12876_2023_3104_MOESM2_ESM.docx]

# SUPPLEMENTARY TABLES

S1 Table. PICOS criteria for SLR.

| **PICOS** | **Inclusion** | **Exclusion** |
| --- | --- | --- |
| Population | - Adult patients (≥18 yrs.) with CC or CIC - CC patients with other disease^1^ | - Patients (Aged ≤18 years) - Patients with OIC and IBS-C - Healthy volunteers |
| Interventions | - Lubiprostone (48 mcg) - Linaclotide (145 mcg or 500 mcg) - Elobixibat (10 or 15 mg) | Interventions other than those listed in the inclusion column |
| Comparators | - Same as interventions - Placebo | Comparators other than those listed in the inclusion column |
| Outcomes | For efficacy:   - SBMs - Stool consistency - Straining severity - Abdominal bloating - Abdominal pain/discomfort - Constipation severity   For safety:   - TEAEs - Gastrointestinal disorders - Diarrhea - Nausea - Vomiting - Cardiovascular - Electrolyte - Metabolic | Outcomes other than those specified in the inclusion column |
| Study design | - RCTs - Observational study (prospective and retrospective) - SLR^2^ | - Conference Abstracts - Case reports - Case series - Animal studies - Letters - Editorials - General reviews - Studies with size ≤100 |
| Time scope | - Time: last 15 years (2005-Current) |  |

^1^CC with other disease includes Parkinson disease, diabetes, cystic fibrosis, non-alcoholic fatty liver disease and hemodialysis.

^2^Relevant literature reviews were excluded and flagged for back reference check.

Abbreviations: CC: Chronic Constipation; CIC: Chronic Idiopathic Constipation; IBS-C: Irritable bowel syndrome with constipation; OIC: Opioid Induced Constipation; RCT: Randomized controlled Trials; SBM: Spontaneous Bowel Movement; SLR: Systematic Literature Review; TEAEs: Treatment emergent adverse events.

S2 Table. NOS for assessing the quality of observational and single arm trial studies.

| **Author, year** | **Selection** | | | | **Comparability** | **Outcome/exposure** | | | **Total quality assessment score & overall study quality** | |
| --- | --- | --- | --- | --- | --- | --- | --- | --- | --- | --- |
|  | **Representativeness of the cases** | **Selection of the control group** | **Ascertainment of exposure** | **Demonstration that outcome of interest was not present at start of study** | **Comparability of subjects in cohort based on the design or analysis** | **Assessment of outcome** | **Was follow-up long enough for outcomes to occur** | **Adequacy of follow-up of cohorts** |  |  |
| Shah, 2020 | 1 | 1 | 1 | 1 | 2 | 1 | 1 | 1 | 9 | High |
| Tomie, 2020 | 1 | NA | 1 | 1 | 2 | 1 | 0 | 1 | 7 | High |
| Eguchi, 2020 | 1 | NA | 1 | 1 | 2 | 1 | 0 | 1 | 7 | High |
| Nakajima, 2018a | 1 | NA | 1 | 1 | 1 | 1 | 1 | 1 | 7 | High |
| Fukudo, 2015 | 1 | NA | 1 | 1 | 1 | 1 | 1 | 1 | 7 | High |
| Lembo, 2011b | 1 | NA | 1 | 1 | 1 | 1 | 1 | 0 | 6 | Medium |
| Abe, 2020 | 1 | NA | 1 | 1 | 1 | 1 | 0 | 1 | 6 | Medium |

Abbreviations: NA: Not applicable; NOS: Newcastle–Ottawa Scale.

S3 Table. Stool consistency and straining severity for lubiprostone, linaclotide, and elobixibat.

| **Author, year** | **Drug** | **Study characteristics** | **Stool consistency** | **Straining severity** |
| --- | --- | --- | --- | --- |
| Eguchi, 2020 | Lubiprostone | - Population: ≥65 yrs. (80%) - Population type: CC - Sample Size: 1,338 - Design: Retrospective observational study - Region: Japan | BSFS score (**7-point scale**: **1-7**), Mean (SD)  Week 2   - Before: 2.6 (1.2) - After: 4.8 (1.1); *p<*0.01 vs Baseline | NR |
| Fukudo, 2015 | Lubiprostone  Placebo | - Population: Adults - Population type: CIC - Sample Size: 124 - Design: RCT, phase-III - Region: Japan | BSFS score (**7-point scale: 1‑7**), (Mean)  Week 1   - Lubi 48 mcg: 3.78; *p<*0.001 vs PBO - PBO: 2.36   Week 2   - Lubi 48 mcg: 3.49; *p<*0.001 vs PBO - PBO: 2.51   Week 3   - Lubi 48 mcg: 3.37; *p=*0.001 vs PBO - PBO: 2.55   Week 4   - Lubi 48 mcg: 3.66; *p<*0.001 vs PBO - PBO: 2.6 | NR |
| Fukudo, 2011 | Lubiprostone | - Population: Adults - Population type: CIC plus IBS-C - Sample Size: 170 - Design: RCT phase-II (dose finding study) - Region: Japan | BSFS score (**7-point scale: 1-7**), P-value  Change from baseline:  Week 1:   - Lubi 48 mcg: *p<*0.0001 vs. PBO   Week 2:   - Lubi 48 mcg: *p<*0.0001 vs. PBO | Straining severity (**5-point scale: 0-4**), P-value  Change from baseline:  Week 1:   - Lubi 48 mcg: p=0.0004 vs. PBO   Week 2:   - Lubi 48 mcg: p=0.0008 vs. PBO |
| Johanson, 2007 | Lubiprostone  Placebo | - Population: Adults - Population type: CC - Sample Size: 127 - Design: RCT; phase-II (dose-ranging study) - Region: USA | - BSFS score (**5-point scale: 1-5**), Mean (SD)   Week-1   - Lubi 48 mcg: 2.4 (0.15); *p=*0.001 vs. PBO - PBO: 3.3 (0.12)   Week-2   - Lubi 48 mcg: 2.6 (0.17); *p=*0.005 vs. PBO - PBO: 3.4 (0.14)   Week-3   - Lubi 48 mcg: 2.7 (0.18); *p=*0.009 vs. PBO - PBO: 3.4 (0.16)   Week-(1-3)   - Lubi 48 mcg: 2.5 (0.13); *p<*0.0001 vs. PBO - PBO: 3.4 (0.11) | - Straining severity score (**5-point scale: 0-4**), Mean (SD)   Week-1   - - - Lubi 48 mcg: 1.2 (0.13); *p=*0.02 vs. PBO     - PBO: 1.6 (0.19)   Week-2   - Lubi 48 mcg: 1.3 (0.14); *p=*0.215 vs. PBO - PBO: 1.8 (0.26)   Week-3   - Lubi 48 mcg: 1.3 (0.15); *p=*0.1 vs. PBO - PBO: 1.9 (0.21)   Week-(1-3)   - Lubi 48 mcg: 1.2 (0.1); *p=*0.005 vs. PBO - PBO: 1.8 (0.17) |
| Fukudo, 2019 | Linaclotide  Placebo | - Population: Adults - Population type: CC - Sample Size: 181 - Design: RCT, phase-III - Region: Japan | Week 1   - BSFS score (**7-point scale: 1-7)**, Mean (95% CI) - Lina 500mcg: 4.12 (3.85, 4.39); *p<*0.001 vs PBO - PBO: 2.87 (2.59, 3.14)   Week 1   - Mean (95% CI) change from baseline in BSFS frequency: - Lina 500mcg: 1.54 (1.27, 1.82); *p<*0.001 vs PBO - PBO: 0.29 (0.02, 0.56) | Week 1   - Straining severity score (**5-point scale: 1-5)**, Mean (95% CI) - Lina 500mcg: 2.30 (2.12, 2.48); *p<*0.001 vs PBO - PBO: 2.81 (2.63, 2.99)   Week 1   - Mean (95% CI) change from baseline in Straining severity score: - Lina500mcg: ‐0.84 (‐1.02, ‐0.66); *p<*0.001 vs PBO - PBO: ‐0.33 (‐0.51, ‐0.15) |
| Fukudo, 2018 | Linaclotide  Placebo | - Population: Adults - Population type: CC - Design: RCT; phase-II (dose‐finding study) - Sample Size: 382 - Region: Japan | Weekly mean stool form score (**7-point scale: 1-7)**:  Week 1   - Lina 500mcg: 4.44; *p<*0.001 vs PBO - PBO: 3.21   Week 2   - Lina 500mcg: 4.41; *p<*0.001 vs PBO - PBO: 3.28   Mean change from baseline in BSFS frequency:  Week 1   - Lina 500mcg: 1.75; *p<*0.001 vs PBO - PBO: 0.52   Week 2   - Lina 500mcg: 1.77; *p<*0.001 vs PBO - PBO: 0.64 | Weekly mean straining severity score (**5-point scale: 1-5)**:  Week 1   - Lina 500mcg: 2.07; *p<*0.001 vs PBO - PBO: 2.78   Week 2   - Lina 500mcg: 2.02; *p<*0.001 vs PBO - PBO: 2.70   Mean change from baseline in Straining severity score:  Week 1   - Lina 500mcg: -1.03; *p<*0.001 vs PBO - PBO: -0.32   Week 2   - Lina 500mcg: -1.10; p <0.001 vs PBO - PBO: -0.42 |
| Schoenfeld, 2018 | Linaclotide  Placebo | - Population: Adults - Population type: CIC - Design: RCT; phase-III, (NCT02291679) - Sample Size: 1223 - Region: USA | Week-12  BSFS score (**7-point scale: 1-7)**, (Mean)   - Lina 145 mcg: 3.7 - PBO: 3   Week-12   - Mean change from baseline in BSFS frequency: - Lina 145 mcg: 1.8; *p<*0.0001 vs PBO - PBO: 1.1 | Week-12  Straining severity score (**5-point scale: 1-5)**, (Mean)   - Lina 145 mcg: 2.3 - PBO: 2.7   Week-12   - Mean change from baseline in Straining severity score: - Lina 145 mcg: -1.2; *p<*0.0001 vs PBO) - PBO: -0.8 |
| Lacy, 2015 | Linaclotide  Placebo | - Population: Adults - Population type: CIC - Design: RCT; phase-IIIb, (NCT01642914) - Sample Size: 483 - Region: USA/Canada | Week-12   - BSFS score **(7-point scale: 1-7**), (Mean) - Lina 145 mcg: 4.3 - PBO: 3.1 - Mean change from baseline in BSFS frequency: - Lina 145 mcg: 1.9; *p<*0.0001 vs PBO - PBO: 0.7 | Week-12   - Straining severity score **(5-point ordinal scale: 1-5**), (Mean) - Lina 145 mcg: 2.2 - PBO: 2.8 - Mean change from baseline in Straining severity score: - Lina 145 mcg: -1.5; *p<*0.0001 vs PBO - PBO: -0.8 |
| Lembo, 2011a | Linaclotide  Placebo | - Population: Adults - Population type: CC - Design: RCT (Trial 303); phase-III; (NCT00730015) - Sample Size: 642 - Region: USA/Canada | Week-12   - BSFS score (7-point scale: 1-7), (Mean) - Lina 145 mcg: 4.3 - PBO: 3 - Mean change from baseline in BSFS frequency: - Lina 145 mcg: 1.9; *p<*0.001 vs PBO - PBO: 0.6 | Week-12   - Straining severity score **(5‑point ordinal scale: 1‑5**), (Mean) - Lina 145 mcg: 2.1 - PBO: 2.7 - Mean change from baseline in Straining severity score: - Lina 145 mcg: -1.1; *p<*0.001 vs PBO - Lina 290 mcg: -1.2; *p<*0.001 vs PBO - PBO: -0.5 |
|  | Linaclotide  Placebo | - Population: Adults - Population type: CC - Design: RCT (Trial 01); phase-III; (NCT00765882) - Sample Size: 630 - Region: USA/Canada | Week-12   - BSFS score (7-point scale: 1-7), (Mean) - Lina 145 mcg: 4.2 - PBO: 2.9 - Mean change from baseline in BSFS frequency: - Lina 145 mcg: 1.8; *p<*0.001 vs PBO - PBO: 0.6 | Week-12  Straining severity score **(5-point ordinal scale: 1-5**), (Mean)   - Lina 145 mcg: 2.1 - PBO: 2.7 - Mean change from baseline in Straining severity score: - Lina 145 mcg: -1.1; *p<*0.001 vs PBO - PBO: -0.6 |
| Tomie, 2020 | Elobixibat | - Population: Elderly - Population type: CC - Design: RCS - Sample Size: 104Region: Japan | Week 2  Elob  Improvement rate in stool consistency (N=136): 59.6% | NR |
| Nakajima, 2018b | Elobixibat  Placebo | - Population: Adult - Population type: CC plus IBS-C - Design: RCT, phase IIb, (JapicCTI-142608) - Sample Size: 163 - Region: Japan | - BSFS score (**7-point** **scale: 1-7**), (Mean)   Week 1   - Elob 10 mg: 4; *p<*0.001 vs PBO - Elob 15 mg: 4.6; *p<*0.001 vs PBO - PBO: 2.9   Week 2   - Elob 10 mg: 4.3; *p<*0.001 vs PBO - Elob 15 mg: 4.6; *p<*0.001 vs PBO   PBO: 3 | NR |
| Nakajima, 2018a | Elobixibat  Placebo | - Population: Adult - Population type: CC plus IBS-C - Design: RCT, phase-III, (JapicCTI-153061) - Sample Size: 132 - Region: Japan | - BSFS score (**7-point scale**), Mean (SD)   Week 1   - Elob 10 mg: 4.3(1.2); *p<*0.0001 vs. PBO - PBO: 2.6(1.1)   Week 2   - Elob 10 mg: 4.3(1.2); *p<*0.001 vs PBO   PBO: 2.9(1.4) | NR |
| Abe, 2020 | Elobixibat | - Design: Retrospective study - Population: CC - Sample Size: 149 - Region: Japan | - BSFS score (**7-point scale**), Mean (SD)   Baseline: 2.5(1.8)  Week 2  Elob 5/10/15 mg: 3.4(1.7); *p<*0.001 vs Baseline | NR |
| Chey, 2011 | Elobixibat | - Population: Adults - Population type: CIC - Design: RCT; phase IIb (NCT01007123) - Sample Size: 190 - Region: USA | - BSFS score (**7-point scale**), Mean (SD)   Change from baseline at week 8:   - Elob 10 mg: P <0.001 - Elob 15 mg: P <0.001 | - Straining severity score (**5-point scale**), Mean (SD)   Change from baseline at week 8:   - Elob 10 mg: P<0.001 - Elob 15 mg: P<0.001 |

Abbreviations: BSFS: Bristol stool form scale; CC: Chronic Constipation; CI: Confidence Interval; CIC: Chronic Idiopathic Constipation; Elob: Elobixibat; IBS-C: Irritable bowel syndrome with constipation; Lina: Linaclotide; Lubi: Lubiprostone; mcg: Microgram; mg: Milligram; NR: Not Reported; PBO: Placebo; RCS: Retrospective Cohort Study; RCT: Randomized controlled Trial; SD: Standard Deviation; USA: United States of America.

S4 Table. Abdominal bloating, pain/discomfort for lubiprostone, linaclotide, and elobixibat.

| **Author, year** | **Drug** | **Study characteristics** | **Abdominal bloating** | **Abdominal pain/discomfort** |
| --- | --- | --- | --- | --- |
| Fukudo, 2011 | Lubiprostone  Placebo | - Population: Adults - Population type: CIC plus IBS-C - Sample Size: 170 - Design: RCT phase-II (dose finding study) - Region: Japan | - Abdominal bloating score (**5-point scale:**0-4), P‑value   Change from baseline:  Week 1:   - Lubi 48 mcg: *p=*0.0006 vs. PBO   Week 2:   - Lubi 48 mcg: *p=* 0.0012 vs. PBO | - Abdominal pain/discomfort score (**5-point scale:**0-4), P-value   Change from baseline:  Week 1:   - Lubi 48 mcg: p=0.0182 vs. PBO   Week 2:   - Lubi 48 mcg: p=0.0048 vs. PBO |
| Johanson, 2007 | Lubiprostone  Placebo | - Population: Adults - Population type: CC - Sample Size: 127 - Design: RCT; phase-II (dose-ranging study) - Region: USA | - Abdominal bloating score (**5-point scale:** 0-4), Mean (SD)   Week-1:   - Lubi 48 mcg: 1.6 (0.18); *p=*0.045 vs. PBO - PBO: 1.8 (0.14)   Week-2:   - Lubi 48 mcg: 1.7 (0.17); *p=*0.264 vs. PBO - PBO: 1.8 (0.15)   Week-3:   - Lubi 48 mcg: 1.5 (0.18); *p=*0.574 vs. PBO - PBO: 1.7 (0.13)   Week-(1-3):   - Lubi 48 mcg: 1.7 (0.16); *p=*0.035 vs. PBO - PBO: 1.9 (0.13) | - Abdominal pain/discomfort score (**5-point scale:** 0-4)**,** Mean (SD)   Week-1:   - Lubi 48 mcg: 1.7 (0.2); *p=*0.140 vs. PBO - PBO: 1.8 (0.16); vs. PBO   Week-2:   - Lubi 48 mcg: 1.3 (0.19); *p=*0.296 vs. PBO - PBO: 1.4 (0.13)   Week-3:   - Lubi 48 mcg: 1.3 (0.16); *p=*0.356 vs. PBO - PBO: 1.7 (0.14)   Week-(1-3):   - Lubi 48 mcg: 1.5 (0.17); *p=*0.136 vs. PBO - PBO: 1.7 (0.13) |
| Barish, 2010 | Lubiprostone  Placebo | - Design: RCT; phase 3 - Population: CC - Sample Size: 237 | - Abdominal bloating score (5-point scale: 0-4), Mean (SD)   Week-1:   - Lubi 48 mcg: 1.44 (1.126); P=0.038 vs PBO - PBO: 1.71 (1.107)   Week-2:   - Lubi 48 mcg: 1.41 (1.050); P=0.6274 vs PBO - PBO: 1.49 (1.079)   Week-3:   - Lubi 48 mcg: 1.50 (1.009); P=0.1788 vs PBO - PBO: 1.71 (1.039)   Week-4:   - Lubi 48 mcg: 1.39 (1.053); P=0.2800 vs PBO - PBO: 1.59 (1.096) | - Abdominal pain/discomfort score (5-point scale: 0-4), Mean (SD)   Week-1:   - Lubi 48 mcg: 1.23 (0.967); P=0.1514 vs PBO - PBO: 1.40 (1.047)   Week-2:   - Lubi 48 mcg: 1.16 (1.056); P=0.8716 vs PBO - PBO: 1.14 (1.042)   Week-3:   - Lubi 48 mcg: 1.34 (0.997); P=0.806 vs PBO - PBO: 1.41 (1.120)   Week-4:   - Lubi 48 mcg: 1.24 (1.018); P=0.1383 vs PBO - PBO: 1.47 (1.168) |
| Fukudo, 2019 | Linaclotide  Placebo | - Population: Adults - Population type: CC - Sample Size: 181 - Design: RCT, phase-III - Region: Japan | Abdominal bloating score (**5‐point ordinate scale**:1-5), Mean (SD)  Week 1: Mean (95% CI)   - Lina 500mcg: 2.00 (1.89, 2.11); *p=*0.835 vs PBO - PBO: 2.02 (1.91, 2.13)   Week 1   - Mean (95% CI) change from baseline in Abdominal bloating score: - Lina 500mcg: ‐0.17 (‐0.28, ‐0.06); *p=* 0.835 vs. PBO - PBO: ‐0.15 (‐0.26, ‐0.04) | Abdominal pain/discomfort score (**5‐point ordinate scale**:1-5), Mean (SD)  Week 1: Mean (95% CI)   - Lina 500mcg: 1.98 (1.87, 2.09); *p=*0.031 vs PBO - PBO: 1.81 (1.70-1.92)   Week 1   - Mean (95% CI) change from baseline in Abdominal pain/discomfort score: - Lina 500mcg: 0.07 (-0.05, 0.18); p= 0.031 vs PBO - PBO: −0.11 (−0.22, 0.00) |
| Schoenfeld, 2018 | Linaclotide  Placebo | - Population: Adults - Population type: CIC - Design: RCT; phase-III, (NCT02291679) - Sample Size: 1223 - Region: USA | Week-12   - Abdominal bloating score (**11‐point NRS: 0-10**), Mean (SD) - Lina 145 mcg: 3.7 - PBO: 4.2 - Mean change from baseline in Abdominal bloating score: - Lina 145 mcg: -1.5; *p<*0.0001 vs PBO - PBO: -1.1 | Week-12   - Abdominal discomfort score (**11‐point NRS: 0-10**), Mean (SD) - Lina 145 mcg: 3.3 - PBO: 3.6 - Mean change from baseline in Abdominal discomfort score: - Lina 145 mcg: -1.4; P = 0.0056 vs PBO - PBO: -1.1 - Abdominal pain score (**11‐point NRS: 0-10**), Mean (SD) - Lina 145 mcg: 2.9 - PBO: 3.2 - Mean change from baseline in Abdominal pain score: - Lina 145 mcg: -1.3; *p=*0.0029 vs PBO - PBO: -1 |
| Lembo, 2010 | Linaclotide  Placebo | - Design: RCT (Dose-range-finding study) - Population: CC - Sample Size: 307 - Region: USA | - Week-4 - Mean change from baseline in Abdominal bloating score (5-point ordinal scale: 1-5): - Lina 75 mcg: -0.40; P≤0.05 vs PBO - Lina 150 mcg: -0.42; P≤0.05 vs PBO - Lina 300 mcg: -0.27; P≤0.05 vs PBO - Lina 600 mcg: -0.26; P≤0.05 vs PBO - PBO: -0.02 | - Week-4 - Mean change from baseline in Abdominal discomfort score (5-point ordinal scale: 1-5): - Lina 75 mcg: -0.32; P≤0.05 vs PBO - Lina 150 mcg: -0.30; P≤0.05 vs PBO - Lina 300 mcg: -0.24; P≤0.05 vs PBO - Lina 600 mcg: -0.28; P≤0.05 vs PBO - PBO: -0.04 |
| Lacy, 2015 | Linaclotide  Placebo | - Population: Adults - Population type: CIC - Design: RCT; phase-IIIb, (NCT01642914) - Sample Size: 483 - Region: USA/Canada | Week-12:   - Abdominal bloating score (**11-point NRS: 0-10**), Mean (SD) - Lina 145 mcg: 4.7 - PBO: 5.5 - Mean change from baseline in Abdominal bloating score: - Lina 145 mcg: -2.5; p=0.0002 vs PBO - PBO: -1.6 | NR |
| Lembo, 2011a | Linaclotide  Placebo | - Population: Adults - Population type: CC - Design: RCT (Trial 303); phase-III; (NCT00730015) - Sample Size: 642 - Region: USA/Canada | - Abdominal bloating score (**5-point ordinal scale: 1-5)**, Mean   Week-12:   - Lina 145 mcg: 2.3 - PBO: 2.5 - Mean change from baseline in Abdominal bloating score: - Lina 145 mcg: -0.5; *p<*0.001 vs PBO - PBO: -0.2 | - Abdominal discomfort score (**5-point ordinal scale: 1-5)**, Mean   Week-12:   - Lina 145 mcg: 2 - PBO: 2.2 - Mean change from baseline in Abdominal discomfort score: - Lina 145 mcg: -0.5; *p<*0.001 vs PBO - PBO: -0.3 |
|  | Linaclotide  Placebo | - Population: Adults - Population type: CC - Design: RCT (Trial 01); phase-III; (NCT00765882) - Sample Size: 630 - Region: USA/Canada | - Abdominal bloating score (**5-point ordinal scale: 1-5)**, Mean (SD)   Week-12:   - Lina 145 mcg: 2.3 - PBO: 2.6 - Mean change from baseline in abdominal bloating score: - Lina 145 mcg: -0.4; *p<*0.001 vs PBO - PBO: -0.2 | - Abdominal discomfort score (**5-point ordinal scale: 1-5)**, Mean (SD)   Week-12   - Lina 145 mcg: 2 - PBO: 2.3 - Mean change from baseline in Abdominal discomfort score: - Lina 145 mcg: -0.5; *p<*0.0001 vs PBO - PBO: -0.3 |
| Abe, 2020 | Elobixibat | - Design: RCS - Population: CC - Sample Size: 149 - Region: Japan | - NR | - Abdominal pain/discomfort score (Constipation Scoring System sub score), Mean (SD)   Mean Score at Baseline:   - Elob 5/10/15 mg: 0.7 (1.3)   Week 2   - Elob 5/10/15 mg: 0.8(1.3); *p=*0.237 vs Baseline |
| Chey, 2011 | Elobixibat  Placebo | - Population: Adults - Population type: CIC - Design: RCT; phase IIb (NCT01007123) - Sample Size: 190 - Region: USA | - Abdominal bloating score (**5-point scale**: 1-5), Mean (SD)   Baseline:   - Elob 15 mg: 2.8 (0.9) - PBO: 2.8 (0.8)   Change from baseline week 8:   - Elob 15 mg: *p=*<0.05 vs. PBO | - NR |

Abbreviations: CC: Chronic Constipation; CI: Confidence Interval; CIC: Chronic Idiopathic Constipation; Elob: Elobixibat; IBS-C: Irritable bowel syndrome with constipation; Lina: Linaclotide; Lubi: Lubiprostone; mcg: Microgram; mg: Milligram; NR: Not Reported; NRS: Numeric Rating Scale; PBO: Placebo; RCS: Retrospective Cohort Study; RCT: Randomized controlled trial; SD: Standard Deviation; USA: United States of America.

S5 Table. Constipation severity for lubiprostone, linaclotide, and elobixibat.

| **Author, year** | **Drug** | **Study characteristics** | **Constipation severity** |
| --- | --- | --- | --- |
| Fukudo, 2015 | Lubiprostone  Placebo | - Population: Adults - Population type: CIC - Sample Size: 124 - Design: RCT, phase-III - Region: Japan | Constipation severity (**5-point scale:** 0-4); Mean (SD)  Week 1:   - Lubi 48 mcg: 1.49 (1.01); P<0.001 vs. PBO - PBO: 2.27 (0.99)   Week 2:   - Lubi 48 mcg : 1.59 (0.95); *p=*0.008 vs. PBO - PBO: 2.10 (1.09)   Week 4:   - Lubi 48 mcg: 1.52 (1.01); *p=*0.031 vs. PBO - PBO: 1.97 (1.22) |
| Fukudo, 2011 | Lubiprostone  Placebo | - Population: Adults - Population type: CIC plus IBS-C - Sample Size: 170 - Design: RCT phase-II (dose finding study) - Region: Japan | Constipation severity (5-point scale: 0-4); Mean  Week 1:   - Lubi 48 mcg: 1.1; *p<*0.0001 vs. PBO - PBO: 2.1   Week 2:   - Lubi 48 mcg: 1.1; *p=*0.0008 vs. PBO - PBO: 1.9 |
| Lembo, 2011 | Lubiprostone | - Design: Single arm trial Prospective open-labeled trial - Population: CIC - Sample Size: 248 - Region: USA | Constipation severity (**5-point scale:** 0-4), Mean (SD)  **Baseline:** 2.94 (0.73)  Post-Treatment: <0.0001 |
| Johanson, 2008 | Lubiprostone  Placebo | - Design: RCT; phase-III - Population: CC - Sample Size: 242 | Constipation severity (5 points scale: 0-4), Mean (SD)  Week 1:   - Lubi 24 mcg: 1.97 (1.16); P≤0.0003 Vs PBO - PBO: 2.49 (1.01)   Week 2:   - Lubi 24 mcg: 1.78 (1.12); P≤0.0003 Vs PBO - PBO: 2.41 (1.01)   Week 3:   - Lubi 24 mcg: 1.91 (1.10); P≤0.0003 Vs PBO - PBO: 2.48 (1.11)   Week 4:   - Lubi 24 mcg: 1.94 (1.20); P≤0.0003 Vs PBO   PBO: 2.52 (1.13) |
| Johanson, 2007 | Lubiprostone  Placebo | - Population: Adults - Population type: CC - Sample Size: 127 - Design: RCT; phase-II (dose-ranging study) - Region: USA | Constipation severity (5-point scale: 0-4), Mean (SD)  Week 1   - Lubi 48 mcg: 1.7(0.23); *p=*0. 21 Vs. PBO - PBO: 2.2(0.16)   Week 2   - Lubi 48 mcg: 1.5(0.20); *p=*0.038 Vs. PBO - PBO: 2.0(0.17)   Week 3   - Lubi 48 mcg: 1.5(0.25); *p=*0.019 Vs. PBO - PBO: 2.2(0.18)   Week 1-3   - Lubi 48 mcg: 1.6(0.20); *p=*0.01 vs. PBO - PBO: 2.2(0.15) |
| Barish, 2010 | Lubiprostone  Placebo | - Population: Adults - Population type: CC - Sample Size: 237 - Design: RCT; phase-III - Region: global | Constipation severity (5-point scale: 0-4), Mean (SD)  Week 1   - Lubi 24 mcg: 1.84 (1.253); *p=*0.0061 vs. PBO - PBO: 2.31 (1.253)   Week 2   - Lubi 24 mcg: 1.64 (1.141); *p=* 0.0243 vs. PBO - PBO: 1.99 (1.047)   Week 3   - Lubi 24 mcg: 1.78 (1.143); *p=*0.0265 vs. PBO - PBO: 2.14 (1.143)   Week 4   - Lubi 24 mcg: 1.71 (1.202); *p=*0.0022 vs. PBO - PBO: 2.22 (1.133) |
| Schoenfeld, 2018 | Linaclotide  Placebo | - Population: Adults - Population type: CIC - Design: RCT; phase-III, (NCT02291679) - Sample Size: 1223 - Region: USA | Week 12:  Mean constipation severity score (**5-point scale: 1-5**)   - Lina 72 mcg: 2.7 - Lina 145 mcg: 2.6 - PBO: 3   Change from baseline, mean   - Lina 72 mcg: -0.9; *p<*0.0001 vs. PBO - Lina 145 mcg: -1; *p<*0.0001 vs. PBO - PBO: -0.6 |
| Lembo, 2010 | Linaclotide  Placebo | - Population: Adults - Population type: CC - Design: RCT; phase IIb, (Dose-range-finding study) (NCT00402337) - Sample Size: 307 - Region: USA | Week 4  Constipation severity (**5-points scale: 1-5**)  Change from baseline, mean   - Lina 75 mcg: -0.78; *p<*0.001 vs. PBO - Lina 150 mcg: -0.89; *p<*0.001 vs. PBO - Lina 300 mcg: -0.88; *p<*0.001 vs. PBO - Lina 600 mcg: -0.95; *p<*0.001 vs. PBO - PBO: -0.17 |
| Lacy, 2015 | Linaclotide  Placebo | - Population: Adults - Population type: CIC - Design: RCT; phase-IIIb, (NCT01642914) - Sample Size: 483 - Region: USA/Canada | Week 12:  Mean constipation severity score (**5-points scale: 1-5**)   - Lina 145 mcg: 2.7 - Lina 290 mcg: 2.6 - PBO: 3.1   Change from baseline, mean   - Lina 145 mcg: -1.3; *p<*0.0001 vs. PBO - Lina 290 mcg: -1.4; *p<*0.0001 vs. PBO - PBO: -0.8 |
| Lembo, 2011a | Linaclotide  Placebo | - Population: Adults - Population type: CC - Design: RCT (Trial 303); phase-III; (NCT00730015) - Sample Size: 642 - Region: USA/Canada | Week 12  Constipation severity (**5-points scale: 1-5**): Mean (SD)   - Lina 145 mcg: 2.3 - Lina 290 mcg: 2.5 - PBO: 3   Change from baseline, mean   - Lina 145 mcg: -0.90; *p<*0.001 vs. PBO - Lina 290 mcg: -0.81; *p<*0.001 vs. PBO - PBO: -0.27 |
|  | Linaclotide  Placebo | - Population: Adults - Population type: CC - Design: RCT (Trial 01); phase-III; (NCT00765882) - Sample Size: 630 - Region: USA/Canada | Week 12  Constipation severity (**5-points scale: 1-5**): Mean (SD)   - Lina 145 mcg: 2.4 - Lina 290 mcg: 2.3 - PBO: 3   Change from baseline, mean   - Lina 145 mcg: -0.91; *p<*0.001 vs. PBO - Lina 290 mcg: -0.95; *p<*0.001 vs. PBO - PBO: -0.31 |
| Nakajima, 2018b | Elobixibat  Placebo | - Population: Adult - Population type: CC plus IBS-C - Design: RCT, phase IIb, (JapicCTI-142608) - Sample size: n - Elob 10 mg: 39 - Elob 15 mg: 41 - Region: Japan | Constipation severity (**5-point scale:** 0-4); Mean (SD)  Week 1   - Elob 10 mg: *p=*0.0224 - Elob 15 mg: *p=*0.0018   Week 2  Elob 15 mg: *p=*0.0172 |

Abbreviations: RCT: Randomized controlled Trial; CIC: Chronic Idiopathic Constipation; IBS-C: Irritable bowel syndrome with constipation; Lubi: Lubiprostone; PBO: Placebo; mcg: Microgram; CC: Chronic Constipation; USA: United States of America; SD: Standard Deviation; Lina: Linaclotide; Elob: Elobixibat; mg: Milligram.

S6 Table. Safety outcomes for lubiprostone, linaclotide, and elobixibat.

| Author, year | Drug | Study Characteristics | Timepoint | AEs % |
| --- | --- | --- | --- | --- |
| Eguchi, 2020 | Lubiprostone | - Population: ≥65 yrs. (80%) - Population type: CC - Sample Size: 1,338 - Design: Retrospective study - Region: Japan | 2-weeks | Diarrhea: 6.1  Nausea: 4.2 |
| Fukudo, 2015 | Lubiprostone  Placebo | - Population: Adults - Population type: CIC - Sample Size: 124 - Design: RCT, phase-III - Region: Japan | 4-weeks | Diarrhea: 14.5  **Nausea**: 14.5  Abdominal pain: 3.2 |
|  | Lubiprostone  Placebo | - Population: Adults - Population type: CIC - Sample Size: 209 - Design: RCT; Phase-III (Long-term safety analysis) - Region: Japan | 48-weeks | Diarrhea: 37.3  **Nausea**: 27.3  Vomiting: 4.8  Abdominal pain: 5.3 |
| Fukudo, 2011 | Lubiprostone | - Population: Adults - Population type: CIC plus IBS-C - Sample Size: - Lubi 48 mcg: 44 - Design: RCT phase-II (dose finding study) - Region: Japan | 2-weeks | Diarrhea:   - Lubi 48 mcg: 18.2   Nausea:   - Lubi 48 mcg: 15.9   Vomiting:   - Lubi 48 mcg: 4.5 |
| Shah, 2020 | Lubiprostone | - Design: Retrospective study - Population: IBS-C plus CIC - Sample Size: 157 - Region: USA | 3-month | Diarrhea: 3.2  **Nausea**: 10.8  Abdominal pain: 7 |
| Lembo, 2011 b | Lubiprostone | - Design: Single arm trial (Prospective open-labeled trial) - Population: CIC - Sample Size: 248 - Region: USA | 48-weeks | TEAEs (%)  Diarrhea: 11.3  Nausea: 21  Abdominal pain: 6 |
| Johanson, 2008 | Lubiprostone  Placebo | - Population: Adults - Population type: CC - Sample Size: 242 - Design: RCT; phase-III - Region: USA | 4-weeks | TEAEs (%);  Diarrhea: 5  **Nausea**: 31.7  Abdominal pain: 5 |
| Johanson, 2007 | Lubiprostone  Placebo | - Population: Adults - Population type: CC - Sample Size: - Lubi 48 mcg: 32 - Design: RCT; phase-II (dose-ranging study) - Region: USA | 3-weeks | Diarrhea:  Lubi 48 mcg: 6.3  Nausea:   - Lubi 48 mcg: 43.8   Abdominal pain:   - Lubi 48 mcg: 3.1 |
| Barish, 2010 | Lubiprostone  Placebo | - Population: Adults - Population type: CC - Sample Size:   Lubi 24 mcg: 119   - Design: RCT; phase-III - Region: Multicentre | 4-weeks | TEAEs (%);  Diarrhea: 3.4  Nausea: 21  Abdominal pain: 6.7 |
| Fukudo, 2019 | Linaclotide  Placebo | - Population: Adults - Population type: CC - Sample Size: 92 - Design: RCT, phase-III - Region: Japan | 4-weeks | Diarrhea  Lina 500mcg: 13  PBO: 5.6  *p=*0.002 |
| Fukudo, 2018 | Linaclotide  Placebo | - Population: Adults - Population type: CC - Design: RCT; phase-II (dose‐finding study) - Sample Size :Lina 500mcg: 76 - Region: Japan | 2-weeks | Diarrhea:  Lina 500mcg: 3.9 |
| Shah, 2020 | Linaclotide | - Design: Retrospective study - Population: IBS-C plus CIC - Sample Size: 128 - Region: USA | 3-month | Diarrhea: 30.5  Nausea: 0  Abdominal pain: 14.1 |
| Schoenfeld, 2018 | Linaclotide  Placebo | - Population: Adults - Population type: CIC - Design: RCT; phase-III, (NCT02291679) - Sample Size: - Lina 145 mcg: 411 - Region: USA | 12-weeks | TEAEs:   - Lina 145 mcg: 35.3   Diarrhea:   - Lina 145 mcg: 22.1 |
| Lacy, 2015 | Linaclotide  Placebo | - Population: Adults - Population type: CIC - Design: RCT; phase-IIIb, (NCT01642914) - Sample Size: - Lina 145 mcg: 153 - Lina 290 mcg: 160 - Region: USA/Canada | 12-weeks | Diarrhea:   - Lina 145 mcg: 5.9   Nausea:   - Lina 145 mcg: 3.9   Vomiting:   - Lina 145 mcg: 3.9   Abdominal pain:   - Lina 145 mcg: 3.9 |
| Nakajima, 2018b | Elobixibat | - Population: Adult - Population type: CC plus IBS-C - Design: RCT, phase IIb, (JapicCTI-142608) - Sample size: n - Elob 5 mg: 43 - Elob 10 mg: 39 - Elob 15 mg: 41 - Region: Japan | 2-weeks | TEAEs:   - Elob 10 mg: 28.2 - Elob 15 mg: 17.1   Diarrhea:   - Elob 10 mg: 5.1 - Elob 15 mg: 7.3   Nausea:   - Elob 10 mg: 2.6 - Elob 15 mg: 0   Vomiting:   - Elob 10 mg: 2.6 - Elob 15 mg: 0   Abdominal Pain:   - Elob 10 mg: 25.6 - Elob 15 mg: 12.2 |
| Nakajima, 2018a | Elobixibat  Placebo | - Population: Adult - Population type: CC plus IBS-C - Design: RCT, phase-III, (JapicCTI-153061) - Sample Size: 69 - Region: Japan | 2-weeks | Elob 10 mg:   - Diarrhea: 13 - Nausea: 3   Abdominal Pain: 19 |
|  | Elobixibat | - Design: Single arm trial - Population: CC plus IBS-C - Sample Size: 340   Region: Japan | 52-weeks | Elob 5-15 mg:   - Diarrhea: 15 - Nausea: 3 - Abdominal Pain: 24 |
| Kumagai, 2018 | Elobixibat | - Population: Adult - Population type: CC   Design: RCT, phase I; (Dose-escalating design)   - Sample Size: - Elob 10 mg: 9 - Elob 15 mg: 10 - Region: Japan | 2-weeks | Diarrhea:   - Elob 10 mg: 44.4 - Elob 15 mg: 40   **Abdominal Pain**: (upper/lower part of abdominal)   - Elob 10 mg:   - Upper: 22.2   - Lower: 33.3 - Elob 15 mg:   - Upper: 0   - Lower: 40 - Elob 20 mg:   - Upper: 20 - Lower: 50 |
| Abe, 2020 | Elobixibat | - Design: Retrospective study - Population: CC - Sample Size: 149   Region: Japan | NR | - Diarrhea: 6 - Nausea: 1.3 - Abdominal Pain: 5.3 |
| Tomie, 2020 | Elobixibat | - Population: Elderly - Population type: CC - Design: RCS - Sample Size: 104 - Region: Japan | 2-weeks | Elob   - Diarrhea: 11.4 - Nausea: 0.7 - Abdominal Pain: 8.6 |
| Chey, 2011 | Elobixibat  Placebo | - Population: Adults - Population type: CIC - Design: RCT; phase IIb (NCT01007123) - Sample size: - Elob 10 mg: 47 - Elob 15 mg: 48 - Region: USA | 8-weeks | TEAEs (%):   - Elob 10 mg: 62 - Elob 15 mg: 65   Diarrhea:   - Elob 10 mg: 6.4 - Elob 15 mg: 12.5   Nausea:   - Elob 10 mg: 4.3 - Elob 15 mg: 6.3   Abdominal Pain:   - Elob 10 mg: 10.6 - Elob 15 mg: 27.1 |

Abbreviations: AEs: Adverse Events; CC: Chronic Constipation; CIC: Chronic Idiopathic Constipation; Elob: Elobixibat; IBS: Irritable bowel syndrome; IBS-C: Irritable bowel syndrome with constipation; Lina: Linaclotide; Lubi: Lubiprostone; mcg: Microgram; mg: Milligram; NR: Not Reported; RCS: Retrospective Cohort Study; RCT: Randomized controlled Trial; TEAEs: Treatment emergent adverse events; USA: United States of America.
